# Identification of small molecular inhibitors of SIRT3 by computational and biochemical approaches a potential target of breast cancer

**DOI:** 10.1038/s41598-024-63177-7

**Published:** 2024-05-30

**Authors:** Atta Ullah, Najeeb Ur Rehman, Waseem Ul Islam, Faizullah Khan, Muhammad Waqas, Sobia Ahsan Halim, Afnan Jan, Abdullatif Bin Muhsinah, Ajmal Khan, Ahmed Al-Harrasi

**Affiliations:** 1 Natural and Medical Sciences Research Center, University of Nizwa, 616 Birkat Al Mauz, PO Box 33, Nizwa, Oman; 2 Department of Pharmacy, University of Swabi, Khyber Pakhtunkhwa, Pakistan; 3 Department of Biochemistry, Faculty of Medicine, Umm Al-Qura University, Mecca, Kingdom of Saudi Arabia; 4 Department of Pharmacognosy, College of Pharmacy, King Khalid University, 61441 Abha, Saudi Arabia

**Keywords:** SIRT3, Breast cancer, Molecular docking, Inhibitor, Flow cytometry, MTT assay, Biochemistry, Cancer, Computational biology and bioinformatics, Drug discovery

## Abstract

Sirtuin 3 (SIRT3) belongs to the Sirtuin protein family, which consists of NAD^+^-dependent lysine deacylase, involved in the regulation of various cellular activities. Dysregulation of SIRT3 activity has been linked to several types of cancer, including breast cancer. Because of its ability to stimulate adaptive metabolic pathways, it can aid in the survival and proliferation of breast cancer cells. Finding new chemical compounds targeted towards SIRT3 was the primary goal of the current investigation. Virtual screening of ~ 800 compounds using molecular docking techniques yielded 8 active hits with favorable binding affinities and poses. Docking studies verified that the final eight compounds formed stable contacts with the catalytic domain of SIRT3. Those compounds have good pharmacokinetic/dynamic properties and gastrointestinal absorption. Based on excellent pharmacokinetic and pharmacodynamic properties, two compounds (**MI-44** and **MI-217**) were subjected to MD simulation. Upon drug interaction, molecular dynamics simulations demonstrate mild alterations in the structure of proteins and stability. Binding free energy calculations revealed that compounds **MI-44** (− 45.61 ± 0.064 kcal/mol) and **MI-217** (− 41.65 ± 0.089 kcal/mol) showed the maximum energy, suggesting an intense preference for the SIRT3 catalytic site for attachment. The in-vitro MTT assay on breast cancer cell line (MDA-MB-231) and an apoptotic assay for these potential compounds (**MI-44**/**MI-217**) was also performed, with flow cytometry to determine the compound’s ability to cause apoptosis in breast cancer cells. The percentage of apoptotic cells (including early and late apoptotic cells) increased from 1.94% in control to 79.37% for **MI-44** and 85.37% for **MI-217** at 15 μM. Apoptotic cell death was effectively induced by these two compounds in a flow cytometry assay indicating them as a good inhibitor of human SIRT3. Based on our findings, **MI-44** and **MI-217** merit additional investigation as possible breast cancer therapeutics.

## Introduction

The SIRT3 gene encodes a protein in humans known as NAD-dependent deacetylase sirtuin-3, mitochondrial (SIRT3). SIRT3 is a mammalian sirtuin, a family of proteins that share sequence similarity with the SIRT2 protein found in yeast^1^. SIRT3 exhibits NAD+-dependent deacetylase activity. SIRT3 is an N-terminally mitochondrial processing peptide-containing soluble protein found in the mitochondrial matrix^2^. When SIRT3 is overexpressed in cultivated cells, both respiration and the generation of reactive oxygen species increase^3^. Since SIRT3 has the highest levels of mitochondrial deacetylase activity, it has garnered a lot of interest^4^. Because of its potential to control the acetylation of numerous mitochondrial proteins—including metabolic enzymes and transcription factors—SIRT3 is crucial to a wide range of cellular processes^5^. Previous research has shown that SIRT3 dysregulation in cellular pathways may enhance carcinogenesis and chemotherapy resistance^6^. Therefore, SIRT3 expression could be used to screen for people at the time of cancer diagnosis who are more likely to develop metastases or drug-resistant illness^7^. Worldwide, cancer remains a serious health issue, with metastasis being a key factor in cancer-related deaths. In cancer, SIRT3 suppresses glycolysis-dependent tumors while promoting oxidative phosphorylation-dependent tumors^8^. Depending on the cell and tumor type, as well as the presence or absence of various stress or cell death triggers, SIRT3 can act as either a tumor promoter or a tumor suppressor^9^. Although SIRT3 has been linked to cancer in certain studies, its utility as a therapeutic target remains debatable. Given the importance of mitochondrial function for cancer, it is interesting that MDA-MB-231 refers to a much-studied human breast cancer cell line lacking the expression of estrogen receptors, progesterone receptors and human epidermal growth factor receptor^10^. MDA-MB-231 cells (metastatic breast adenocarcinoma), derived from a pleural effusion of a patient with an invasive ductal carcinoma at M D Anderson, are commonly used to recapitulate advanced stages of breast cancer^11^. MDA-MB-231 research has not only helped to reveal the molecular basis of triple-negative breast cancer but also may provide a useful tool to reveal the part SIRT3 plays in cancer pathways^12,13^. As a result, a unique approach to treating cancer will be much easier to achieve if we have a better grasp of the mechanisms underlying the disparate cancer kinds^14^. As compared to healthy cells, cancer cells produce more reactive oxygen species (ROS)^15^. Mutations in DNA sequence and other metabolic pathways involved in tumor cell proliferation and transformation can be influenced by reactive oxygen species^2^. A modest increase in ROS may be beneficial to cell survival and proliferation; however, an excessive accumulation of ROS may cause cell death. As a result, ROS's function is still up for debate^16^. Short non-coding RNAs known as microRNAs (miRNAs) have a role in controlling the expression of specific genes post-transcriptionally. Evidence suggests a link between miRNA dysregulation and carcinogenesis^17^. SIRT3 has been reported to bind miR-1225-5p (miRNA). Thyroid cancer cells grow, proliferate and metastasized faster if miR-1225-5p is downregulated and miR-224 is upregulated. Overexpression of miR-224 may mediate similar effects in non-small cell lung cancer cells by directly targeting SIRT3^18^. MiR-1225-5p downregulation and miR-224 overexpression may increase tumor cell growth, proliferation, and metastasis in thyroid cancer and non-small cell lung cancer cells, respectively, by directly targeting SIRT3^19,20^. The carcinogenic mechanism of SIRT3 has been the subject of several research. A changed phenotype is generated *in-vitro* and *in-vivo* by the IDH2K413 acetylation mimic, which reduces mitochondrial respiration and detoxification and raises ROS levels^21^. As a result, many scientists are interested in finding ways to use small-molecule anti-cancer therapies that specifically target SIRT3. The reprogramming of cellular energy metabolism is a defining feature of cancer^22^. SIRT3 is involved in most of these cancer pathways, making it a promising new therapeutic target. The use of in-silico methods is rapidly expanding in the pharmaceutical industry^23,24^. The in-silico drug design process alters the timeline of drug discovery and development by allowing for the rapid identification of new therapeutic candidates^24,25^. Structure-based drug development has advanced to a higher level of originality and efficacy^26^. In the current study, novel inhibitors of SIRT3 were identified from our in-house database by using extensive computational techniques and these compounds are validated by in-vitro and flow cytometry assays..

## Methodology

### SIRT3 protein crystal coordinates

The RCSB protein data repository served for getting the X-ray crystallographic structure of SIRT3 associated with the small antagonist EX527 (https://www.rcsb.org/)^27^ with (PDB ID: 4BVH, and 1.90 Å resolution)^28^. The SIRT3 lacking residues within the (3D) three-dimensional structure were generated through the Loop modeler technique in Molecular Operating Environment (MOE) version 2022.02^29^ using Amber14 EHT (Amber ff14SB combined with EHT)^30^ forcefield. The Quick prep model of MOE 2022.02^31^ was utilized to provide the required hydrogens and to denote the start and end (C-N) terminals of protein residues. Moreover, the van der Waals, bond formation, chirality of residues, missing atom types, and lack of angle and forcefield characteristics were all corrected^32^.

### Screening of inhibitors by docking

The MOE Dock algorithm was put to work to position the chemicals into the desired protein's active pocket, named SIRT3^33^. The crystal structure of human SIRT3 with attached inhibitor EX527was used as a reference during docking. Throughout our investigation, this structure provided a useful baseline. Initially “screening by docking protocol” was validated by enrichment analysis. For this purpose, seven known inhibitors (3-typ ((3-(1H-1,2,3-triazol-4-yl) pyridine)^34^, A939572^35^, AGK2^36^, Nicotinamide^37^, nitro indole, Quinoline-4-carboxylic acid, and Tenovin^35,38,39^) were selected, and their physicochemical descriptors were calculated through MOE-Descriptor calculator. The molecular weight [g/mol] of seven compounds are in range of 122–455, their xlogP _o/w_ values ranges from − 0.38 to 4.88, their net charge is in range of − 0.94 to 0.999, rotatable bonds = 1–13, topological polar surface area (Å^2^) = 50–106, they possess number of hydrogen bond donor atoms in range of 1–3 and number of hydrogen bond acceptors atoms = 1–4. These descriptors were given to ZINC database to search compounds that match with these descriptors, along with polar desolvation [kcal/mol] = − 400.00 to 1.00 and a polar desolvation [kcal/mol] = − 100.00 to 40.00 which is ZINC default parameter. Afterwards, we received 5128 compounds from ZINC database, among which 700 compounds were randomly selected as decoys for our enrichment analysis. Later eight known inhibitors and 700 decoys were prepared as a library and docked by “screening by docking protocol” into 4BVH and enrichment factor and % enrichment was estimated by following formulas:

$${\text{Enrichment}}\;{\text{ Factor }}\left( {{\text{EF}}} \right) = \frac{{\frac{{{\text{hits }}\;{\text{sampled}}}}{{{\text{hits}}\;{\text{ total}}}}}}{{\frac{{{\text{N }}\;{\text{sampled}}}}{{{\text{N }}\;{\text{total}}}}}}$$

$$\% {\text{ EF }} = {\text{ Enrichment }}\;{\text{Factor }} \times { 1}00/{\text{Ideal }}\;{\text{Enrichment}}\;{\text{ Factor}}$$

Success was proclaimed when 50% of eight known inhibitors were ranked in the top 1% (top 8 compounds), 5% (top 36 compounds) and 10% (top 71 compounds) of screened library. The optimal threshold for virtual screening accuracy was justified by the following criteria: If in top 1%, in top 5% and in top 10% of screened database, all the 7 known inhibitors are successfully identified then, the ideal EF would be 100, 20 and 10, respectively. We observed that in 1% of screened library, none of the known inhibitors were identified, therefore at this cut-off, the EF and %EF is zero. While in top-5%, 1 inhibitor was retrieved, which reflect the EF and % EF of 2.45 and 12.25%, respectively. Moreover, at the top-10% of screened library, seven out of eight known inhibitors were successfully identified, which made the EF and %EF of 8.75 and 87.5%, respectively. These results reflect that the selected protocol can identify known inhibitors embedded in a set of decoys, thus can be applied for screening purposes. First, the “screening by docking approach” was applied to screen an in-house database of ~ 800 compounds among which some compounds are isolated from natural products in NMSRC, University of Nizwa (Oman), some are their derivatives, while some compounds are from synthetic origin (Supplementary file 2). London dG scoring algorithm^29^ was used to choose 100 poses, and the Triangle Matcher position set of rules^29^ flipped bonds to place every ligand inside the active pocket. The poses were selected using the London dG scoring function. According to the GBVI-WSA dg grading procedure, the final 30 poses for each compound were determined through^3^ Eq. (1)

1 $$\begin{array}{*{20}c} {G = c + E_{flex} + \mathop \sum \limits_{h - bonds} c_{HB} f_{HB} + \mathop \sum \limits_{m - lig} c_{M} f_{M} + \mathop \sum \limits_{atom i} \Delta D_{i} } \\ \end{array}$$

In this equation ‘c’ is the change in rotational or translational entropy; ‘Eflex’ is the loss of entropy due to loss of flexibility of the ligand; ‘fHB’ is the geometric imperfection factor of hydrogen bonds and varies from 0 to 1; ‘CHB’ is the energy for the existing hydrogen bond; and ‘fM’ is the geometric imperfection factor of the metal ligands, which also varies from 0 to 1. ‘CM’ is the optimal energy for metal binding; and ‘Di’ is the desolation energy of atom ‘i’. The London dG scoring was used to calculate the basis of energy difference between ligand conformation and molecular foundation and obtained the top 30 conformers with the most negative energy. The top 30 ligand conformations were then linearly optimized using Eq. (2) with the GBVI/WSA dG scoring, which is performed in the Molecular Operating Environment (MOE) version 2022.02, to obtain the binding free energies for each ligand conformation. K.

2 $$\begin{array}{*{20}c} {\Delta G \approx c + a\left[ {\frac{2}{3}\left( {\Delta E_{coul} + \Delta E_{sol} } \right) + \Delta E_{vdw} + \beta \Delta SA_{weighted} } \right]} \\ \end{array}$$

In Eq. (2), “c” stands for the mean increase or loss of rotational or transitional entropy, while “a” and “b” are constants that have been established throughout training and are force-field-dependent. The coulombic electrostatic term is called Ec_oul_, while the solvation electrostatic term is called E_sol_. E_vdw_ is the binding contribution of van der Waals. Surface area weighted by exposure is known as SA_weighted_.

### Protein–protein networking of SIRT3

A network pharmacology study of SIRT3 would be aimed at the molecular interactions underlying cancer and mitochondrial stress regulation as responses to pharmacological treatments^40^. To find all the main protein (SIRT3) interactions we analyzed target associated interactions with STRING server. STRING (*Search Tool for the Retrieval of Interacting Genes/Proteins*) is a commonly used database for protein–protein interaction networks^41^. It can present known and predicted protein–protein interaction networks and can be used to interpret and model protein function as well^42^. The nodes in the STRING network presents proteins, and edges present protein–protein associations, and the system scoring algorithm measures a lot of information routes and the results of interactions between proteins depending on the number of paths it can follow to obtain them^43^. At the end of imputations, we can specify a minimum interaction score (0.7 minimum threshold level) that will filter information, just as we did for transcription factors, and keep only high-confidence information^44^. Finally, we exported the Protein–Protein Interaction network and proceeded to perform the statistical analysis of the protein–protein interactions.

### Docking interaction analysis

**Figure 1:**
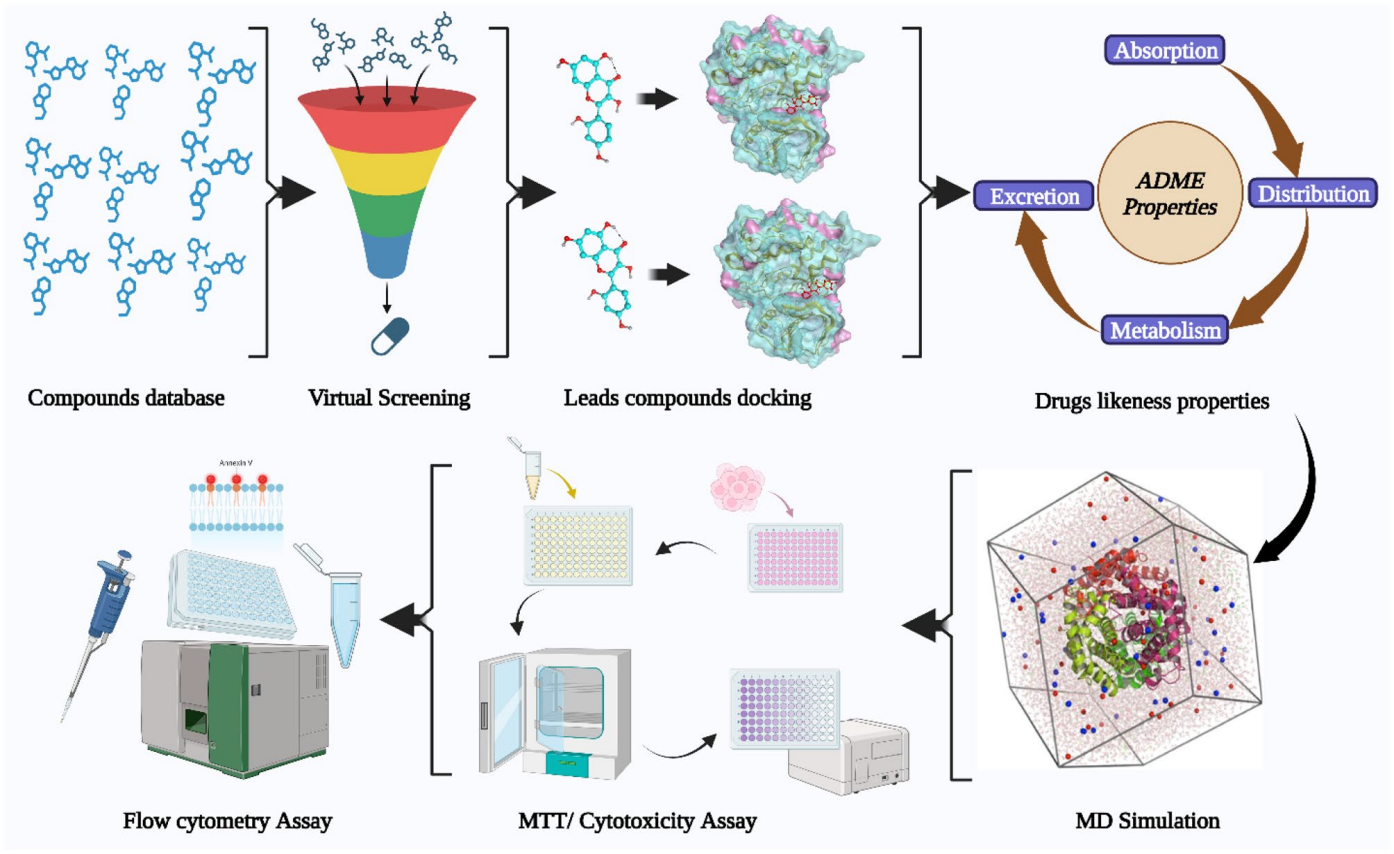
Schematic workflow of the current study design.

Molecular docking, a powerful and growingly important method in rational drug design, facilitates in-silico screening. Docking is the computational process of assessing the energetic and geometric compatibility of several entities like protein and ligand, to identify a ligand that can bind to the protein. After docking of compounds in the SIRT3 binding pocket, the docking score was subsequently utilized to rank the docked collection, and the complexes at the highest ranks were taken as promising ligands. To check the efficacy of docking, re-docking was performed using the co-crystallized ligand of SIRT3 to ensure the docking optimization. Later, conformational sampling was conducted to identify the optimal binding modes of the selected compounds. At the catalytic domain of SIRT3, we used MOE's Protein–Ligand Interaction Fingerprints (PLIF)^29^ to quantify the interactions of molecules. The schematic workflow of this study is presented in Fig. 1.

### Pharmacokinetics and drug-likeness properties calculation

To investigate the chemicals' absorption routes, toxicity profiles, metabolic pathways, and distribution, SwissADME^45^ server was employed which estimates the physiochemical properties and pharmacokinetics of compounds along with their drug-like characteristics.

### Molecular dynamic simulations

In the present work, MD simulations were performed on complexes containing SIRT3 ligands. We picked a reference-inhibited system to perform MD simulation i.e., SIRT3 paired with the inhibitor EX527(PDB ID: 4BVH). The structural modifications imposed by inhibitor binding were compared with APO SIRT3 protein. Molecular dynamics simulations were performed for each system using AMBER22 software package^46^. The Leap module of the AMBER22 was used to generate topology and coordinate files for each system. The residue-specific force field ff19SB was used to parameterize the protein residues and to represent protein–ligand interactions^47^. To every system, monovalent OPC ions (ion Na+ and ion Cl−) were created/added. The divalent OPC ion library was used to parameterize the NI++ elements of the systems, to maintain the electrostatic neutrality among the systems^48^. To obtain well founded simulation results, the system needs to be neutralized by the counterions ligand interactions, the fact that water molecules perform a worthy role in hydration and the stability of simulation should be considered. To mimic the octahedral OPC-water model, the structure was considered for the hydration of the systems. A water buffer box was set up to 12 Å distance from the protein external side. The minimization process was carried out prior to starting the MD production run. At this stage, the energy of system atoms was minimized by a two-step energy minimization run to obtain an energy at the lowest energy, which assisted in the system de-stressing and reduction of steric conflict. In the first step of minimization, the steepest descent technique, an insight into a gradient-based optimization approach, was carried out for 50,000 cycles.^49^. This process is repeated iteratively, changing the atomic positions at each step until the negative potential-energy gradient is obtained. For the steepest descent minimization, which started our first iteration, 25,000 iterations were performed in the conjugated gradient approach^46^. The alternative optimization strategy that takes only a handful of iterations by using information from previous iterations which is called the conjugate gradient method. During these processes, we have kept the water molecules set free for movement, and the protein residues being still. This is useful while stabilizing the protein–ligand complex. We have spread out the water molecules around the protein. As soon as we have reached the final protein–ligand boundary, we need to warm up the systems slowly to the designated temperature and pressure followed a thermostat where we have heated up the systems from a lower temperature to the temperature of the simulation (300 K) over a span of 400 ps^46^. With the Langevin thermostat, system heating is maintained by forcing the stochastic forces upon them, which acts as a surrogate heat bath. During the heating process, the scaling of the kinetic energy was tailored to ensure the propagating dynamics, with the Langevin thermostat having a collision frequency 2.0/ps for the harmonic oscillators. This helps to achieve the required temperature in the system. Before starting dynamic production run, all potential steric clashes were fixed by SHAKE algorithm^49^. SHAKE serves as a constraint algorithm to stabilize the covalent bonds between hydrogen atoms, appropriately extending the run time of the simulation. After the heating phase, the system density was equilibrated by a 400 ps run in that consistent practice. This leaves H_2_O molecules to flow surrounding the protein and ligand complex and completes the preparation of the system for the goal temperature. Next, every system was equilibrized at 300 K (in the NPT ensemble where N = number of particles, P = pressure, T = temperature) for 5000 ps (MD time steps). During this period, there were no restraints on the possible array of molecules and the system was allowed to sample all possible phases that might be present. The final stage of the experiments involved production MD run (main part of the simulation where important motion and or interaction of protein–ligand molecules can be monitored to extract important information). Each system is run for 300 ns in the production run^50^. This is the appropriate length of time to capture the behavior of the protein–ligand complex and obtain meaningful statistical information^51^. This is justification for using broad-well Langevin dynamics, with a cutoff of 8 Å for the non-bonded terms, which was done to accelerate the calculation of interactions that require additional computational resources. These interactions are not computed beyond 8 Å because, although that limits the accuracy of including these interactions, it enables the system to be run more efficiently. At several time points (every 10 ps) during the production run, the atomic positions, and velocities of the relevant parts of the protein–ligand complex are written to a trajectory, which is used to analyze the system’s dynamics.

### MD production evaluation

We used CPPTRAJ, a module available with AMBER 22, to carry out analysis of the simulated trajectory cut at every 1 p^52^. RMSF and RMSD have being assessed for entirely systems operating the Cα atoms based on Eqs. (3, 4).

3 $$\begin{array}{*{20}c} {RMSD = \sqrt {\frac{{\mathop \sum \nolimits_{i = 0}^{N} \left[ { m_{i} *\left( {X_{i} - Y_{i} } \right)^{2} } \right]}}{M}} } \\ \end{array}$$

In RMSD, the number of atoms is “N”, the mass of atoms is “mi”, the target atom vector coordinate is “Yi”, the reference atom vector coordinate is X, and the total mass is M.

4 $$\begin{array}{*{20}c} {RMSF\left( i \right) = \sqrt { \left( {x_{i} - x_{i} } \right.)^{2} } } \\ \end{array}$$

For combining the atom positions in incoming frames x, the RMSF for the Atom i that is relevant is determined. Rg Radius of Gyration is calculated by calculating the position and velocity of atoms in each time step^53^.

### Principal component analysis

The slow movements of the SIRT3 protein that were of interest were determined by the principal component analysis (PCA) functioned operating the CPPTRAJ component of the AMBER 22 software. In Eq. (5), C is the predicted covariance matrix with Cij as its components.

5 $$\begin{array}{*{20}c} {C_{ij} = \left( {x_{i} } \right. - \left. {x_{j} } \right)\left( {x_{i} } \right. - \left. {x_{j} } \right)} \\ \end{array}$$

The ^x^i and ^x^j are the ith and jth Cartesian coordinate of the C atoms of the molecule, and ^x^i and ^x^j the average coordinates of the ith and jth atoms along the ensemble time respectively. The covariance matrix is constructed based on the 3-D spatio-temporal parameters of all production trajectory consisting of the 10 different motions observed. Principal Components (PCs) are summarized by calculating the covariance matrix C. This matrix is diagonalized to obtain eigenvalues and eigenvectors (these quantities describe the features of the motion). Eigenvalues reflect the power of each eigenvector to describe features, which can be quantified, and they tell us something about the relative importance of each description. These PCs are the visual representations of the changes taking place. The graphs below represent PC1 and PC2. To visualize these changes, PC1 and PC2 were displayed on a graph.

### Characterization of hydrogen bonds

For this analysis, we employed the hbond function in CPPTRAJ which was used to examine the bonds between SIRT3 and the ligands. By examining the 300 ns path, we were able to determine an average life span, location, and tilt of the hydrogen bonds that link ligands and the target’s surface. Here, the bond angle between electron acceptor and donor atoms of protein–ligand was set to 120°, and the cutoff distance was fixed at 3.5 Å^54^.

### Binding free energy calculations

We have utilized the generalized born solvation accessible surface area (MMGB-SA) approximation that was implemented in the AMBER22 package to gauge the actual uniting free energies of the preferred ligands in the active situate of the human SIRT3^55^. The free energy and free APO form of the SIRT3 complex were obtained using Eq. (6).

6 $$\begin{array}{*{20}c} {\Delta G_{bind} = G_{R + L} - \left( {G_{R} + G_{L} } \right)} \\ \end{array}$$

The joined vitality of the protein and the inhibitor, indicated by “GR+L,” and the free vitality of authoritative, assigned by “Gbind,” is represented by Eq. (7).

7 $$\begin{array}{*{20}c} {\Delta G = E_{bond} + E_{VDW} + E_{elec} + G_{GB} + G_{SA} - TS_{S} } \\ \end{array}$$

In Eq. (7) below I show the different components of the total energy ‘ΔG’, as applied in calculating the energy of the protein in interaction with and without the cofactor. Ebond van der Waals energy related to bond and dihedral angles Eelec electrostatic energy GGB polar solvent, typically water GSA non-polar solvent, typically water T absolute temperature of both systems SS entropy of the protein.

### Data analysis

All graphs and structures were made using MOE 2022.02 and Blender, and Origin-Pro was used to extract the ensemble of lowest energy structures for each system, which were then used to generate all the graphs.

### Cytotoxicity assay of compounds on Breast Cancer cell line

The cytotoxicity profile of each molecule was assessed using the MTT assay, which involves the utilization of a yellow tetrazolium salt known as 3-(4, 5-dimethylthizol-2-yl)-2, 5- diphenyl tetrazolium bromide. This assay was conducted on the MDA-MB-231 cell line, which is recognized as a suitable model to study triple-negative breast cancer in both in vitro and in vivo settings^11^. The 3T3-L1 fibroblast cell line was utilized as a control in the investigation. The cells were grown in Dulbecco's Modified Eagle Medium (DMEM) supplemented with 10% fetal bovine serum (FBS) and 1% antibiotics, specifically 100 U/mL penicillin. The cells were introduced into a 96-well plate with a concentration of 1.0 × 104 cells per well and subjected to incubation for a duration of 24 h at a temperature of 37 °C in an environment containing 5% carbon dioxide. The media was disposed of, and subsequent to this, both cell lines were subjected to various concentrations (2.5 μM, 5 μM, 10 μM, and 20 μM) of the compounds^56^. After 48 h of incubation 20 μL of MTT solution (5 mg/mL) was pipetted into each well and incubated for another 4 h. Formazan precipitate was dissolved in DMSO after the medium was thrown away. A microplate reader was used to measure the absorbance of solutions at 570 nm. The cytotoxicity was calculated as a percentage of cell viability compared to untreated control cells, and all tests were conducted in triplicate^56^

8 $$\begin{array}{*{20}c} {\% Viability = \frac{Absorbance \;of \;sample}{{Absorbance \;of \;control}} \times 100} \\ \end{array}$$

### Annexin V-FITC/PI double-staining analysis by flow cytometry

The Annexin V-FITC/PI double staining technique was employed to assess the apoptotic status of the cells. This was accomplished using the Annexin V-FITC apoptosis staining/detection kit (ab14085)^57^. To detect early apoptosis, late apoptosis, and necrosis induced by the compound, MDA-MB-231 (1 × 10^6^ cells/dish) was added to a 6 cm dish and treated for 48 h at 37 °C in 3 mL of culture medium containing test compounds at final concentrations of 0, 5 and 15 μM. The MDA-MB-231 (1 × 10^5^) were then stained for 10 min at room temperature with FITC-conjugated Annexin V-FITC and PI in a Ca^2+^-enriched binding buffer (Annexin V-FITC kit) and analyzed by a FACScan flow cytometer^58^. Annexin V-FITC and PI emissions were detected in the FL 1 and FL 2 channels of a FACScan flow cytometer, using emission filters of 525 and 575 nm, respectively. The Annexin V-FITC-/PI- population was regarded as normal healthy cells, while the Annexin V-FITC + /PI- cells were taken as a measure of early apoptosis, Annexin V-FITC+/PI+ as late apoptosis, and Annexin V-FITC-/PI + as necrosis^59^. Approximately 1 × 10^4^ counts were made for each sample and the percentage of distribution of normal, early apoptotic, late apoptotic, and necrotic cells was calculated using FlowJo_v10.9.0 software^58^.

## Results and discussion

### Structure retrieval of human SIRT3

**Figure 2:**
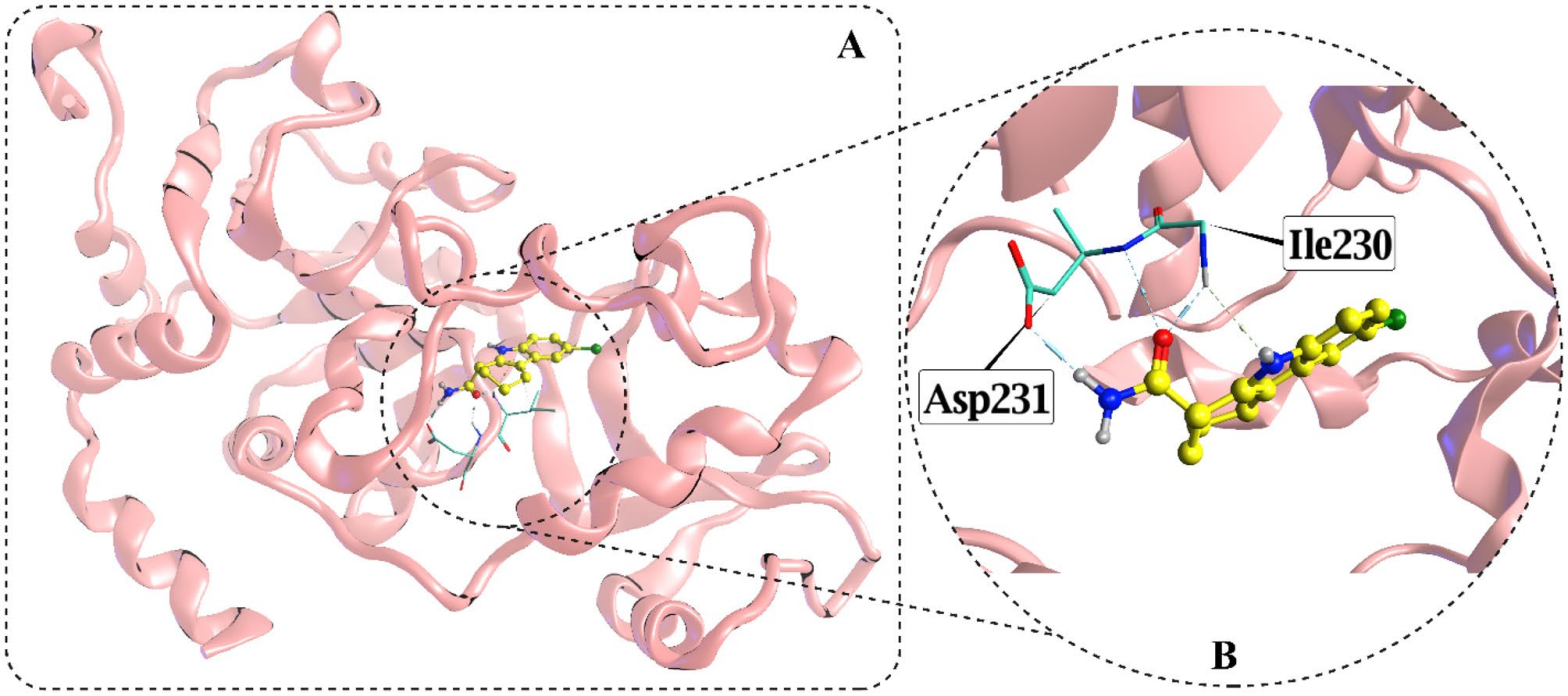
Cartoon representation of SIRT3 catalytic domain with EX527attached in the active pocket.

In the reported human SIRT3 structure, strong hydrogen bonds are formed between active site residues of SIRT3 and EX527inhibitor. The reported inhibitor for SIRT3 has a docking score in the range of − 6.6739 kcal/mol. The residues ASP231 (2×) and ILE230 (3×) interact with the ligand’s acetamide group, thus keeping the drug/ligand in the catalytic region stable (Fig. 2). Moreover, pyrrole nitrogen also forms H-bond with the Ile230. Drug-like compounds in the NMSRC database that target these residues or mimic the binding mechanism of EX527were explored. The complete interaction of the previously available inhibitor (EX527) with the active pocket of SIRT3 is tabulated in Table S1.

### Screening by docking

**Figure 3:**
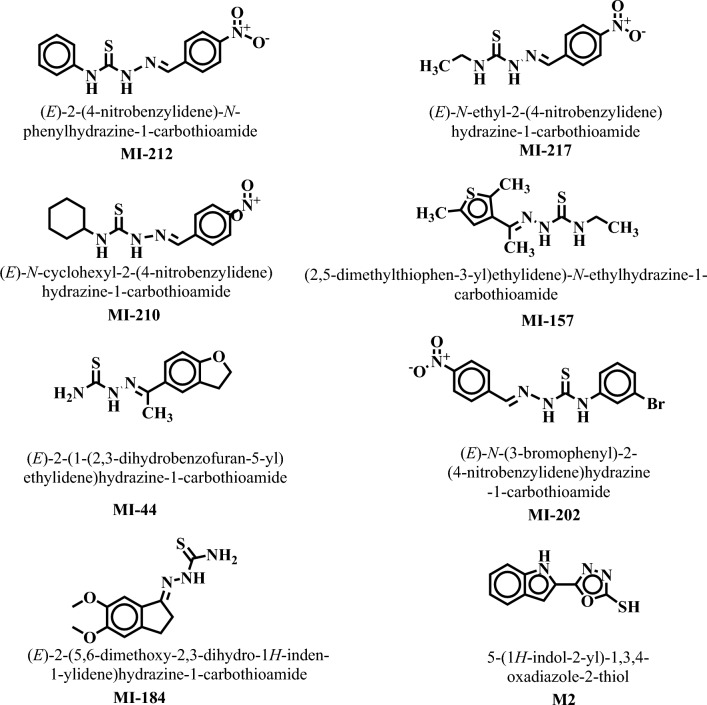
The chemical structures of eight selected compounds are shown.

Before screening our database, we analyzed the capacity of used protocol in the identification of known inhibitors by enrichment analysis. We observed that in 1% of screened libraries, none of the known inhibitors were identified, therefore at this cut-off, the EF and %EF is zero. While in top-5%, 1 inhibitor was retrieved, which reflect the EF and % EF of 2.45 and 12.25%, respectively. Moreover, at the top-10% of screened library, seven out of eight known inhibitors were successfully identified, which made the EF and %EF of 8.75 and 87.5%, respectively. These results reflect that the selected protocol can identify known inhibitors embedded in a set of decoys, thus can be applied for screening purposes. A database of natural products and synthetic compounds was docked in SIRT3 to obtain novel and putative inhibitors of SIRT3. Approximately 800 compounds were virtually screened by docking them into the active pocket of human SIRT3, and compounds with docking scores between − 9 and − 7 kcal/mol were selected from the docked library (Supplementary file 2). A total of 40 top-ranked compounds were selected based on good docking scores. In these top-listed compounds, 8 molecules were further selected based on their good interaction with SIRT3 (Fig. 3). The identified compounds are synthetic compounds of thiosemicarbazones that are published in our previous articles^60,61^.

### SIRT3 targets analysis in the protein–protein interaction (PPI) network

**Figure 4:**
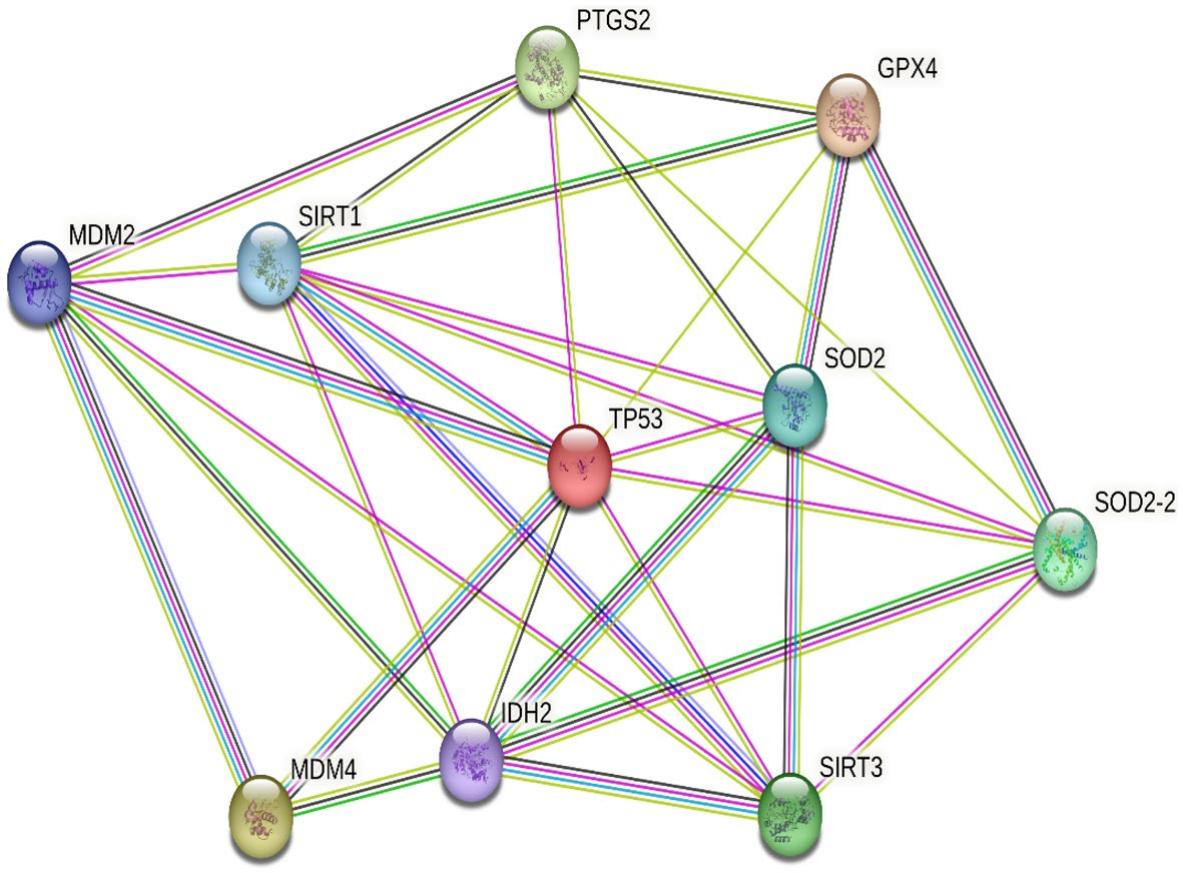
The critical for P53 mediated ferroptosis upon ROS-induced stress PPI network of SIRT3 obtained from STRING server. The color of the nodes represents the degree of binding between proteins.

The protein–protein interaction (PPI) mechanisms were analyzed by creating a PPI map of targeted disease processes (critical for P53mediated ferroptosis upon ROS-induced stress) using STRING server. The PPI's intended disease pathway map for SIRT3 in humans. Target interactions between proteins consisted of 31 edges and 10 nodes (protein–protein association). The average node degree value of these shared targets, as shown by the network analyzer, is 6.2, suggesting that SIRT3 exhibits many targets. Three cluster pathways surrounding SIRT3, included one set of molecular interactions. The first cluster encapsulates 5 nodes, the 5 targetable proteins (MDM2, MDM4, PTGS2, SIRT1, and TP53). The second cluster integrates 4 nodes, the 4 targetable proteins (IDH2, SIRT3, SOD2, and SOD2-2). The third cluster captures a single node, the targetable protein GPX4. In these pathways the core central protein is p53. Since SIRT3 is believed to inhibit p53 activity, it could potentially shut down an apoptotic pathway that would otherwise eliminate damaged cells. The resulting genomic instability could increase the likelihood of these cells developing cancer. The network pharmacology diagram of SIRT3 with interrelated proteins are depicted in (Fig. 4) and Combine K-mean calculated score and nodes interaction tabulated in (Table S2).

### Docking interaction of selected compounds

**Figure 5:**
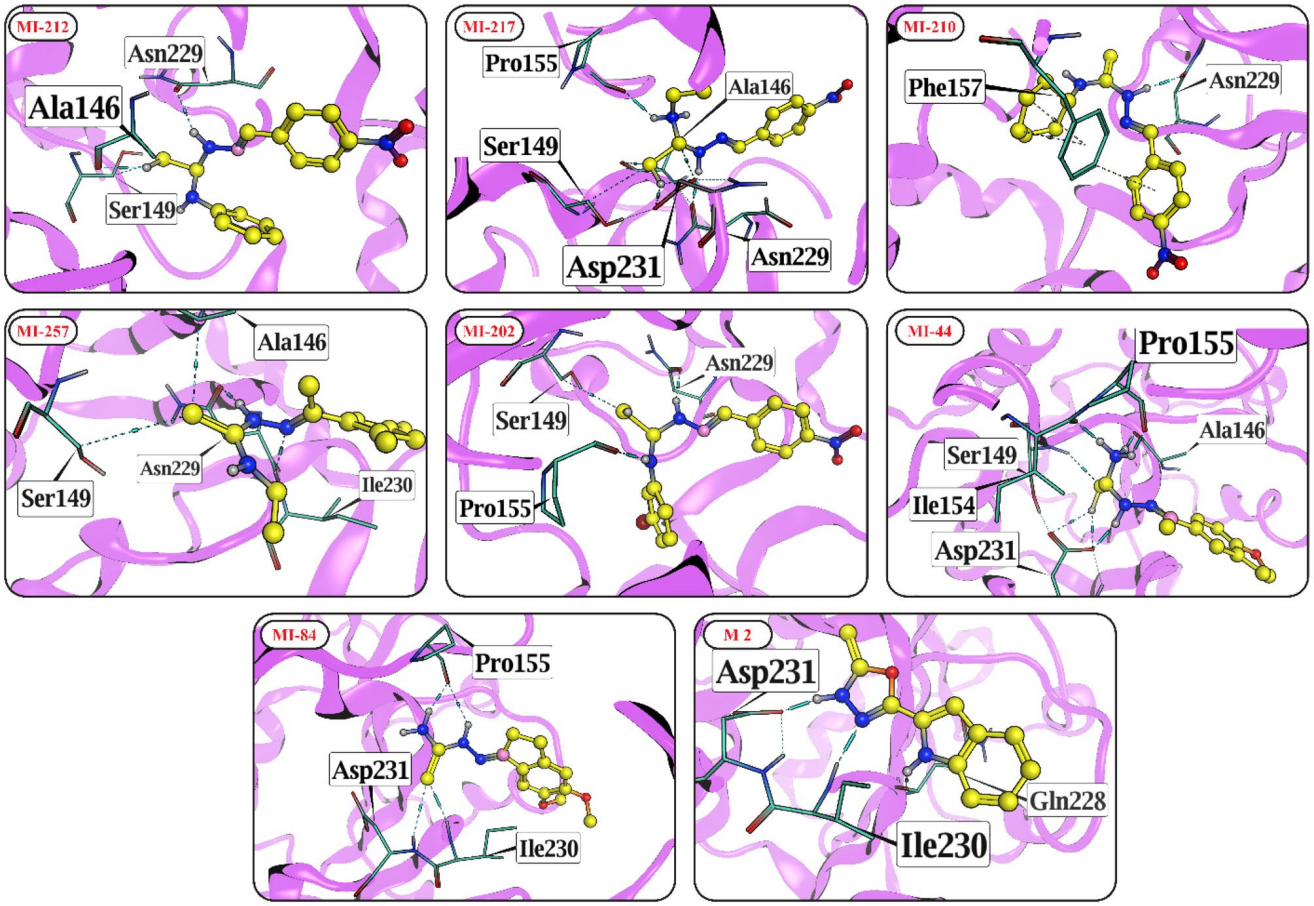
The molecular interaction of selected compounds is shown in the active site of SIRT3. Hydrogen bonds are shown in cyan dashed lines, and the ligands are illustrated in the yellow ball and stick model.

The docking interactions of the finalized eight compounds were visualized by MOE and measured with SIRT3 in the protein complex. We validated our docking by performing a re-docking of the co-crystallized ligands into the functional pocket of SIRT3. The RMSD value amongst the co-crystallized ligands and re-docked inhibitor is 0.927 Å with a docking score of − 7.1442 kcal/mol. These values indicate the orientations of compounds, measured experimentally can be successfully predicted by docking, which justifies the reliability of this docking protocol (Fig. S1). In the selected compounds, the highest docking score is exhibited by **MI-212** (− 8.37 kcal/mol) followed by **MI-217, MI-210, MI-257, MI-202, MI-44, MI-184** and **M2** which exhibits docking score of − 8.15 kcal/mol, − 8.06 kcal/mol − 7.95 kcal/mol, − 7.93 kcal/mol, − 7.48 kcal/mol, − 7.44 kcal/mol and − 7.31 kcal/mol, respectively. We observed that **MI-212** is stabilized through hydrogen bonds with residues ASN229, ALA146, ASP231, and SER149 of SIRT3, while **MI-217** makes hydrogen bonds with ASN229, ASP231, ALA146, PRO155, ASN229 and PRO155. **MI-210** formed a hydrogen bond with ASN229 and a π-H bond with PHE157, thereby stabilizing the protein complex. Moreover, **MI-257** is fixed in the active pocket of the protein through several hydrogen bonds with ASN229, ILE230, ALA146, and SER149. Similarly, **MI-202** and **MI-44** mediates multiple hydrogen bonds with ASN229/PRO155/SER149, and ASP231/ALA146 (2×), respectively. Furthermore, **MI-84** interacts with residues PRO155 (2×) and ILE230 through hydrogen bonds, and **M2** is stabilized in the active site by making hydrogen bonds with GLN228, ASP231, and ILE230 and one π-H bond with HIS248. Thus, by forming several H-bonds and π-H bonds with SIRT3 active site residues, these eight molecules show strong binding affinity for human SIRT3 with minimal energy. The docking interaction of selected compounds is listed in Table S3 and displayed in Fig. 5.

### Drug-likeness, and ADME properties of selected compounds

Analysis of the powerful drug molecule demands in-silico predictions of molecular and physicochemical characteristics, pharmacokinetics, and bioavailability in the context of drug discovery. Here, we present a summary of the in-silico predictions of various characteristics for the selected compounds (Tables S4 and Table 1). The molecular weight (MW) of selected anti-SIRT3 compounds ranged from 217 to 306 g/mol, as determined by an analysis of their substantial molecular and physical features. Topological polar surface area (TPSA) predictions vary from 91.73 to 114.33 Å^2^. The selected inhibitors possess 1–3 hydrogen bond acceptors (HBAs) while donating a maximum of 2 hydrogen bonds. In addition, all compounds fit the rules of Lipinski Ghose and Muegge’s rules of drug-likeness. The lipophilicity (Log P_o/w_ = 1.90–3.10) and water solubility (Log ESOL = − 3.11 to − 4.85) scores predict that the compounds are very soluble in water and suggest that they have moderate to low solubility in non-polar solvents. The selected molecules are expected to be well absorbed in the digestive tract, and none of the compounds showed permeability to oppose the blood–brain barrier (BBB). None of the chemicals had any inhibitory effect on P-glycoprotein (P-gp). Almost all the drug candidates inhibited the cytochrome p450 enzymes (CYP2C19, CYP1A2, CYP2C9, CYP3A4 and CYP2D6). In all these eight selected compounds, **MI-44** and **MI-217** followed the pharmacokinetic and drug-like properties with no blood–brain barrier penetration with good gastrointestinal absorption, therefore, the MD modelling was used to better improve and fine-tune the interactions of two chemicals with SIRT3.

**Table 1:** Physiochemical characteristics of particular substances **(MI-212-M2)**.

Compounds	M/W (g/mol)	NRB	HBA	HBD	TPSA (Å^2^)	Log Po/w	Log (ESOL)	Log Kp (skin)
**MI-212**	300.34	6	3	2	114.33	2.26	− 4.85	− 4.62 cm/s
**MI-217**	252.29	6	3	2	114.33	2.03	− 2.40	− 6.56 cm/s
**MI-210**	306.38	6	3	2	114.33	2.61	− 3.60	− 5.87 cm/s
**MI-257**	255.40	5	1	2	96.75	3.10	− 3.01	− 5.96 cm/s
**MI-202**	379.23	6	3	2	114.33	2.61	− 4.52	− 6.00 cm/s
**MI-44**	235.31	3	2	2	91.73	2.34	− 2.28	− 6.72 cm/s
**MI1-84**	265.33	4	3	2	100.96	1.90	− 2.36	− 6.91 cm/s
**M2-cdx**	217.25	1	3	1	93.51	2.11	− 3.11	− 6.16 cm/s

M/W, molecular weight; NRB, number of rotatable bonds; HBA, hydrogen bond acceptor; HBD, hydrogen bond donor; TPSA, topological polar surface area; Log Po/w, partition coefficient; Log (ESOL), water solubility; Log Kp, skin permeability.

### Molecular dynamic simulation analysis

#### Protein stability analysis (RMSD)

**Figure 6:**
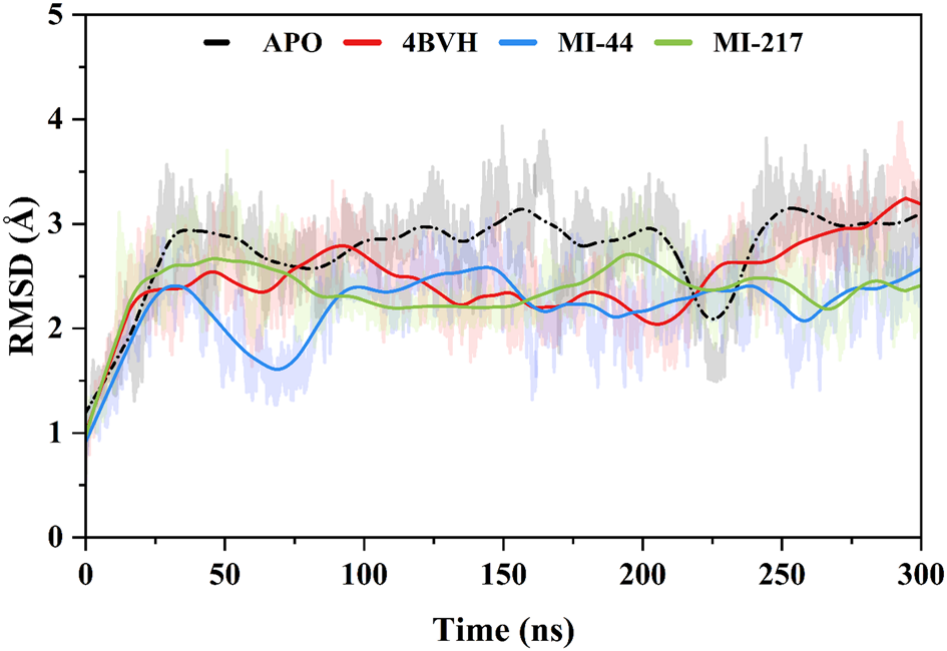
(RMSD) study of the SIRT3 (4BVH) assured with EX527and unbound (APO) with the indicated inhibitors ligands (**MI-44** and **MI-217**).

The APO form of SIRT3 was used to detect structural changes after interaction with the predicted inhibitors. To facilitate the process of docking, AMBER22 was used to simulate the interactions between SIRT3 and the selected compounds. Root-mean-square deviation (RMSD) was evaluated based on the results to find out the change degree of the compounds- from their original form. For the SIRT3 enzyme (4BVH) the average RMSD was 2.49 ± 0.002 Å. All through the simulation at 45 ns and 94 ns a small fluctuation was observed showing an increase of 2.54 Å and 2.78 Å respectively. In the simulation the protein stays stable, and from 280 to 286 ns the value increases to 3.11 Å with fluctuations from 2.78 to 3.00 Å. At the end of the simulation, it got slightly more stable, minimizing movement and showing the protein stayed unchanging. The average RMSD of the protein in APO state i.e. RMSD_APO is 2.77 ± 0.002 Å. At 34 ns there was a slight fluctuation value i.e. 2.9345 Å and again at 158 ns and 254 ns value reached to 3.1352 Å and 3.1458 Å (AMORE.04). After this the protein remains stable for the rest of the simulation. The compound **MI-44** exhibited an average RMSD of 2.22 ± 0.002 Å. However, a small rise at 32–37 ns was observed of 2.41 Å. The compound was stable all through the entire simulation while a small fluctuation was observed at 146 ns (2.57 Å). For compound **MI-217** a small fluctuation was observed at 56 ns (2.5301 Å) and 196 ns (2.7041 Å) at the beginning of the simulation then it gets stable at the end with an average RMSD value of 2.37 ± 0.002 Å. The high fluctuation was observed at the C terminal lobe in the 300 ns MD simulation. The protein stability analysis of compounds is shown in Fig. 6. The simulation trajectory was visually inspected where the C-terminal of the protein moves freely as an elongated loop which contributes to the system RMSD as shown in the supplementary videos 1–4.

#### Analyzing residues fluctuations (RMSF)

**Figure 7:**
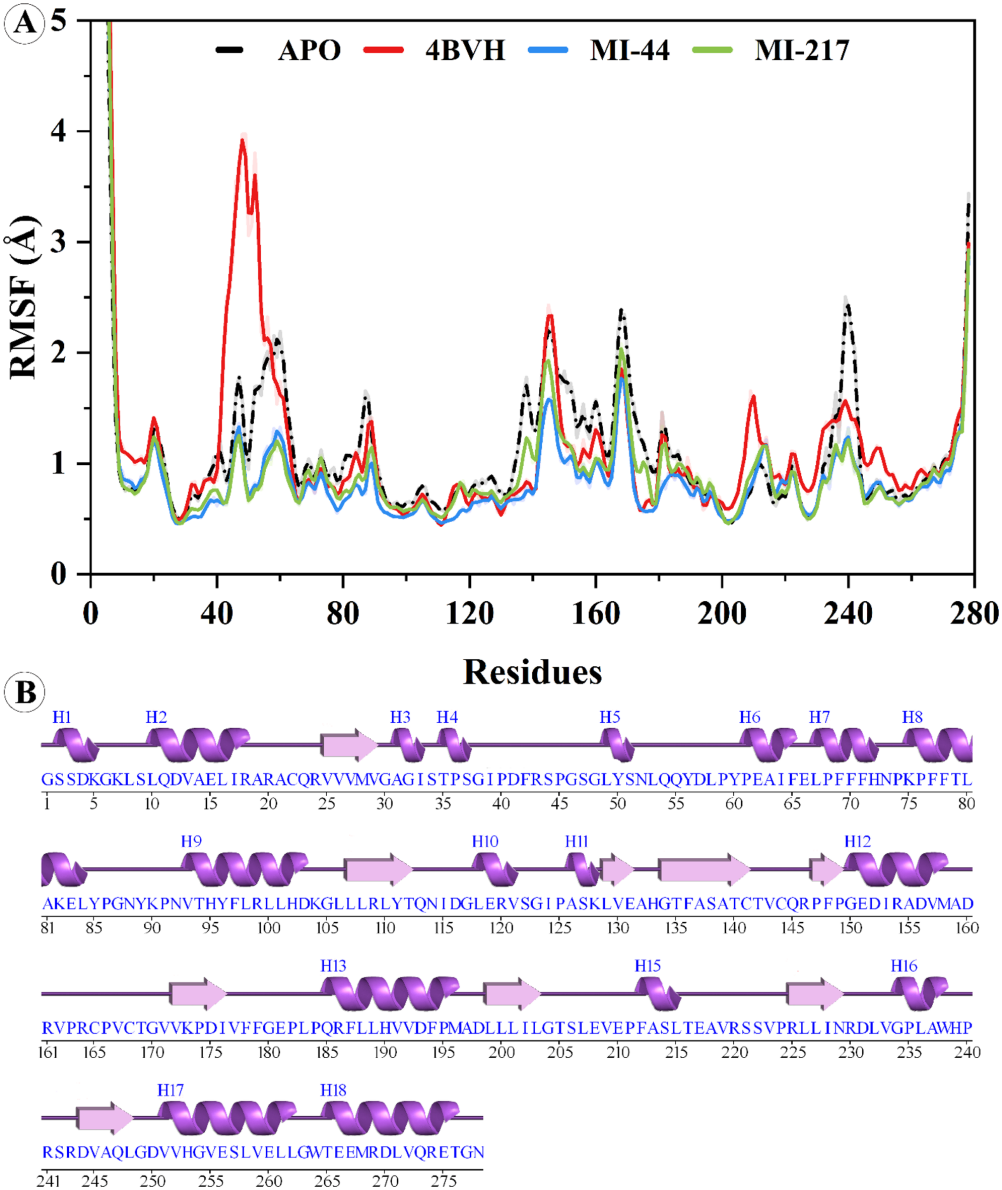
Analyzing SIRT3 in its boundless (APO) form, the reference protein complex (4BVH) bound, and **MI-44** and **MI-217** bound form via root mean square fluctuation.

Root mean square fluctuation (RMSF) is a measure that provides information about the average deviation of an atom from the mean position in the original structure of the protein. In this study, RMSF analysis was performed in the SIRT3 complex with **MI-44** and **MI-217** to evaluate fluctuations of the two compounds in the protein upon attachment. Higher RMSF values of the individual atoms after attachment of the compound denote greater structural flexibility, which is a good condition of later interactions with other inhibitors molecules. Comparing the binding agent (inhibitor) to the protein, the SIRT3 apo-protein had a lower mean RMSF value, with an RMSF value of 1.23 ± 0.074 Å. For the APO form of SIRT3, the average RMSF value is 1.18 ± 0.064 Å. In the APO form, at the start of the simulation, residues from the 52-64 (loop) region fluctuated about 2.15 Å then a small change observed from the 139-157-164 (loop) region of 2.31 Å. The protein became stable at the end of the simulation while a small change was acquired by residues 236-243 (2.3861 Å) at the loop region of the protein The reference inhibited system 4BVH system shows high fluctuation of 3.92 Å in the 44-52 residues which is particularly a long loop region and effected by the ligand attachment which moves in the course of the simulation. The **MI-44** shows an average RMSF value of 1.96 ± 0.007 Å. The protein after the compound binding was almost stable during the entire simulation while alteration was observed at residues 142-149-165-173 (loop) region with fluctuation > 1 Å on average. Similarly, the **MI-217** shows an average RMSF value of 1.02 ± 0.065 Å. After binding to protein, the residues at loop region 143-147-167–173 show a small fluctuation of > 1.5 Å on average. The dynamic motions of these inhibitors and reference complex differed from the apo-state because of the uniting of little inhibitors. The inhibitor-bonded residues showed substantial changes early in the simulation, while the rest of the protein behaved steadily. The established binding of inhibitor chemicals to the active pocket of the SIRT3 is depicted in Fig. 7.

### Evaluation of protein compactness

**Figure 8:**
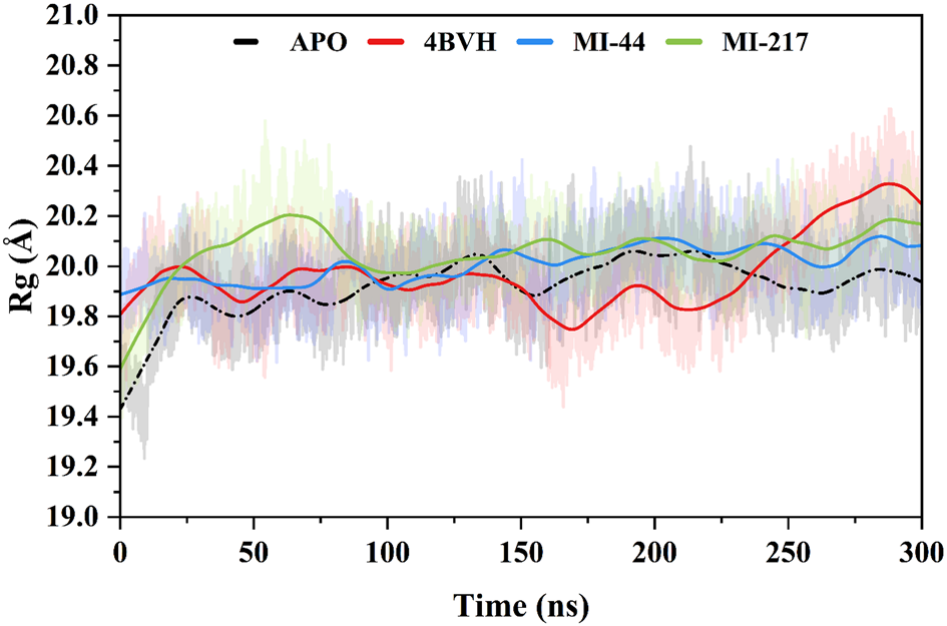
The complex form of human SIRT3 (PDB ID: 4BVH), the free state (APO), and the two chosen inhibitors (MI-44 and MI-217) are shown in a radius of gyration (Rg) plot.

It is important for determine the radius of gyration (Rg) because it provides a simple picture of an object's overall form and assists in predicting how it will react in various circumstances, including factors such as its responsiveness to external factors, potential for preserving and transferring energy, and steadiness. The Rg value for the APO form of SIRT3 is 19.92 ± 0.812 Å. Conversely, a fluctuation was observed at 25–133 ns (20.05 Å) and 192 ns (20.05 Å) then became stable. Likewise, the protein-EX527complex (4BVH) displayed an average Rg value of 19.98 ± 0.001 Å. However, alteration was observed at residues at 18 ns (19.99 Å) and 260 ns (20.19 Å). The dynamic motions of both the selected inhibitors and the reference ligand complex differed from their apo-state counterparts because of the binding of small inhibitors. The average radius of gyration (Rg) value of **MI-44** was 20.01 ± 6.87 Å. At the start of the simulation, a fluctuation of (20.09 Å) average was observed at 8 ns, 143 ns, and 211 ns while the protein became stable at the end. Similarly, the MI-217 at the start of the simulation elucidates a small fluctuation at 96 ns (20.20 Å) then remains stable during the simulation showing an average Rg value of 20.06 ± 0.74 Å (Fig. 8).

#### Principle component analysis

**Figure 9:**
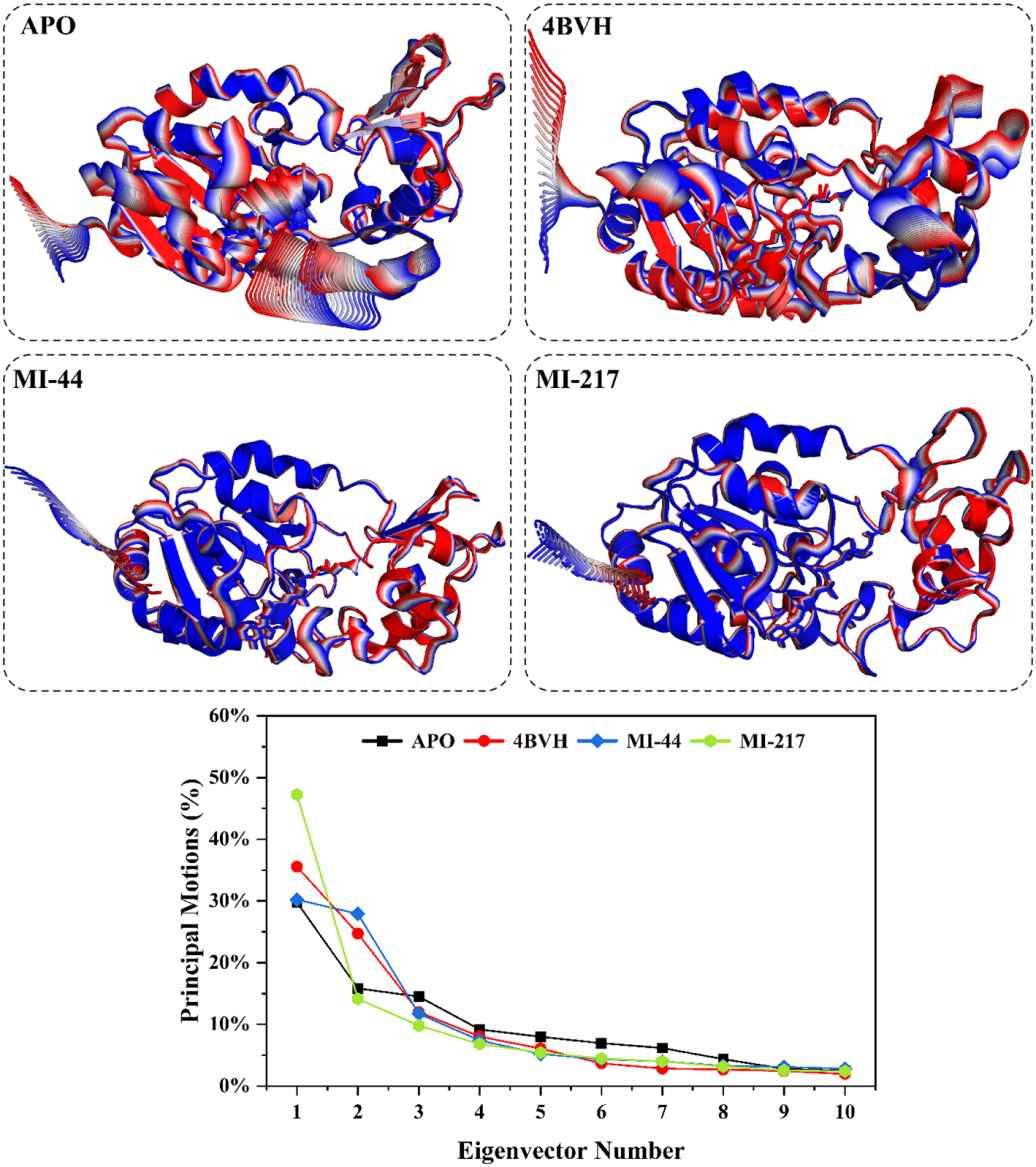
The principal movements displayed by the SIRT3 protein during the 300 ns simulation are revealed by principal component analysis (PCA). Every system was portrayed in a cartoon style, with color changes from blue to red serving as visual cues for changes. For every system, the 2D graph showed different colored eigenvector motions expressed as a percentage.

Principal component analysis (PCA) was performed on the Cα atom trajectory of the SIRT3 protein during a 300 ns simulation trajectory to determine the main motion in the system. Eigenvalues of the covariance matrix were determined and used to calculate the principal components or eigenvectors, from which the overall mobility of the protein was estimated. Ten eigenvectors were calculated for each system to obtain an idea of the main moving parts associated with the dominant modes that describe most structural changes observed over the simulation Fig. 9. In the 300 ns time scale simulation the highest motion was shown by inhibitor **MI-217** which was about 49%. The protein complex 4BVH shows the 2nd highest motion of about 36% as compared to the ligand MI-217. Movement was detected at the site of protein contact, at the top and bottom of B side, where enormous conformational changes were observed. The internal sheets showed the conformational changes upon binding the inhibitors. Similarly, the ligand **MI-44** shows the movement up to 31% followed by the APO free form of protein complex which is about 30%. The structural data highlights the crucial need of focusing on the portions of the SIRT3 surface exhibiting high mobility or conformational changes for efficient inhibition through protein dynamics or structure, and it may also aid in the design of therapeutics.

**Figure 10:**
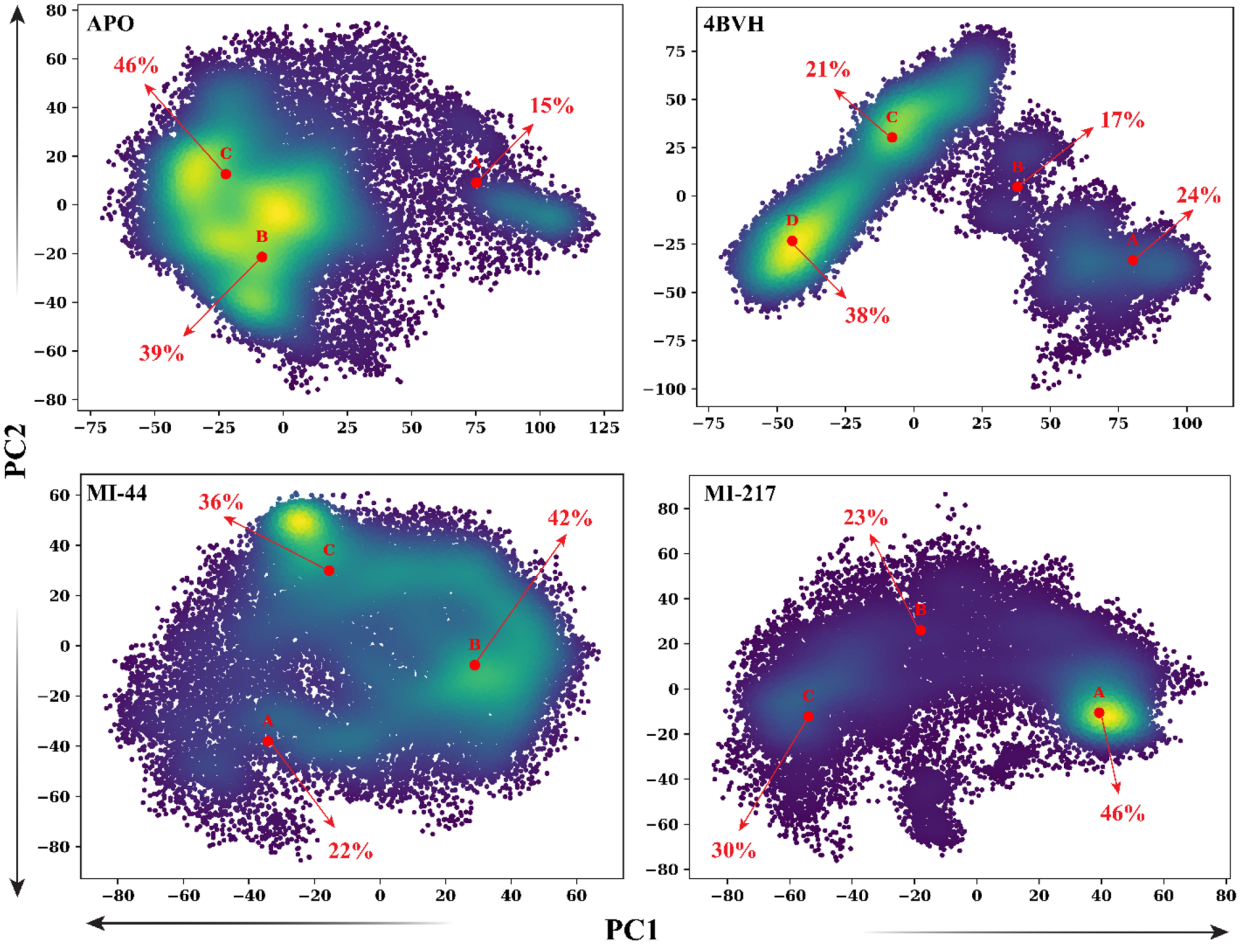
Principal component analysis was performed on the two docked complexes, which included SIRT3 proteins and small molecule inhibitors, as well as on the free state of the proteins and their complexes (4BVH), using a 300 ns simulation duration.

The structural configuration of the SIRT3 protein switched between activated and inhibited state during the 300 ns simulation. This alteration was described by the plot of PC1 and PC2 values plotted against each other Fig. 10. The APO form of protein has shown the confirmation at cluster A of 15% stability then shifted to the cluster B where show the behavior of 39% and finally stable at the cluster C showing stability of 40%. The conformational shift was observed for the SIRT3 protein complex (4BVH). The simulation system spent 24% at cluster A and 17% at cluster B then shifted to the cluster C were spent 21% and the stabilized at cluster D at the end of simulation and stayed about 38%. The ligand **MI-44** stayed at cluster A for 22% then shifted to cluster B for 42% and at the end of simulation became stable and stayed at cluster C for 36%. Similarly, the inhibitor **MI-217** shows notable differences at clusters A (46%) and B (23%) and turn into constant at the last of simulation showing a difference at cluster C of 30%. The activated system 4BVH displays accelerated movement between conformational clusters, eventually stabilizing in the final cluster. In contrast, trajectories of inhibitor-bound systems behave differently from the unbound Apo- and system 4BVH activated systems, occupying different subsets in each, demonstrating that the inhibitor is redirecting the native SIRT3 protein’s dynamics.

#### Hydrogen bond analysis

Hydrogen bonds are essential in maintaining the structure and function of biological molecules. In molecular dynamic simulations, they play a crucial role in determining the dynamics and stability of molecular systems. Hydrogen bond formation between SIRT3 and chosen inhibitors was the focus of the present analysis (Fig. S2 and Table S5). The reference inhibitor (4BVH) makes strong hydrogen bonds with Ile116, Ser207, Thr20 (2×), Asp117, Ser207 (3×), Val252, Arg44 (13×), Leu208, Gln114 (3×), Asn230 (4×), Thr36 (4×) with a bond life span of 91.24%, 75.38%, 63.27%, 63.24%, 46.07%, 33.74%, 25.23%, 23.90%, 20.78%, 16.43%, 13.04% and 12.61%, respectively. The supportive atoms that provide ligand with endurance in SIRT3 active pocket are Asp232 (4×), Ala32, Asp117, Arg231, Tyr51, Arg44 (2×), Arg231, Asp251 (3×), and His134 (2×) with a bond life of < 10% occupancy. The **MI-44** form a strong hydrogen bonds with several residues including Ser207(3X), Ala32, Phe43, Thr206 (2×), Asn230 (3×), Tyr51 (2×), Val252, Arg44, Asn115 and Gln114 which process bond life span of 97.01%, 74.80%, 63.44%, 50.52%, 48.15%, 47.78%, 46.51%, 43.39%, 43.25%, 42.70%, 42.18%, 38.66%, 38.38%, 29.41%, 21.96%, 18.62%, 15.88%, 5.79%, 15.65%, 14.86% and 12.46%, respectively. Moreover, Thr36 (3×), Asp232 (6×), Asp251 (2×), Pro41, Hid134, Gln114, and Arg231 (4×) support the binding of **MI-44** by making hydrogen bonds with a bond life of less than 10%. The compound **MI-217** forms H-bonds with Arg44 (4×), Thr206, Ser207, Ala32, Ser20, Tyr510, Asn230, Phe43, Asn115, Arg44, Gln114, Val252, Asn230 and Thr36 with a bond life span of 97.51%, 92.46%, 89.54%, 81.45%, 66.09%, 61.19%, 50.86%, 49.44%, 48.94%, 47.67%, 45.70%, 41.57%, 38.60%, 16.99%, 13.01%, 11.50% and 11.04%, respectively. Whereas Asn230, Pro41, Asp117, Ser207, Asp117, Thr36O, Asp251 (2×), Arg44N, Asp117 (2×), Gln114, Arg31 (3×), Thr206, Asp251, Asp232 (2×), Ser207 keep up the ligand interaction in active pocket of protein with a bond life span < 10% occupancy. Analyzing hydrogen-bond interactions revealed that the selected ligand chemicals indeed engage in strong hydrogen-bond interactions with the active pocket of human SIRT3, thus making the complex stable for the entire duration of the MD production run.

#### Binding free energy calculation

To ascertain the binding set free energies among distinct predicted inhibitors and SIRT3, the Molecular Mechanics and Generalized Born Solvent Accessible Surface Area (MM-GBSA) approach has been used. The present methodology is employed for the determination of the energy related to chemical substances attaching themselves to a protein’s active site. The utilization of estimated binding free energy holds significance in the field of drug design due to its ability to furnish insights into the intensity of the association between a protein and a binding ligand. This, in turn, facilitates the evaluation of the binding affinity of prospective compounds. Here, the van der Waals energy (ΔE_VDW_), the electrostatic energy (ΔE_EEL_), the polar solvation energy (ΔE_GB_), the non-polar solvation energy (ΔE_SURF_) and the total binding free energy (ΔG_TOTAL_) on inhibitors bound to human SIRT3 (4BVH) were measured. The binding free energy of protein 4BVH (ΔG^TOTAL^) is − 31.55 ± 0.066 kcal/mol with a reduced surface area of − 3.90 Å. The total ΔE_VDW_ energy is (− 35.47 ± 0.041 kcal/mol), ΔE_EEL_ (− 27.94 ± 0.066 kcal/mol), and ΔE_GB_ (35.77 ± 0.068 kcal/mol). The **MI-44** exhibited a high ΔG_TOTAL_ energy i.e., − 45.61 ± 0.064 kcal/mol with ΔE_VDW_ energy of − 51.13 ± 0.046 kcal/mol, ΔE^EEL^ of − 24.55 ± 0.060 kcal/mol and ΔEGB of 36.20 ± 0.039 kcal/mol with a diminished surface area (− 6.13 Å). While **MI-217** exhibited the ΔG_TOTAL_ of − 41.65 ± 0.089 kcal/mol with a demoted apparent area (− 5.56 Å). Also, the ΔE_VDW_ energy for **MI-217** = − 38.71 ± 0.046 kcal/mol, ΔE_EEL_ = − 41.80 ± 0.018 kcal/mol, and ΔE_GB_ = 44.41 ± 0.014 kcal/mol. Table 2 shows the binding energy for these predicted inhibitors.

**Table 2:** The calculation of the binding free energy of specific inhibitors compounds and reference protein (4BVH).

Complex	MM-GBSA xalculations (Unit’s kcal/mol) Differences (receptor–ligand–complex)				
	ΔE_VDW_	ΔE_EEL_	ΔE_GB_	ΔE_SURF_	ΔG_TOTAL_
4BVH	− 35.47 ± 0.041	− 27.94 ± 0.066	35.77 ± 0.068	− 3.90 ± 0.003	− 31.55 ± 0.066
**MI-44**	− 51.13 ± 0.046	− 24.55 ± 0.060	36.20 ± 0.039	− 6.13 ± 0.002	− 45.61 ± 0.064
**MI-217**	− 38.71 ± 0.046	− 41.80 ± 0.018	44.41 ± 0.014	− 5.56 ± 0.001	− 41.65 ± 0.089

ΔE_EEL_, electrostatic energy; ΔE_VDW_, van der Waals energy; ΔG_TOTAL_, total binding free energy; ΔEGB, polar solvation energy; ΔE_SURF_, the nonpolar component of the solvation energy.

#### Cytotoxicity assay

Human breast cancer cell line (MDA-MB-231) was tested with **MI-44** and **MI-217** at doses of 2.5 μM, 5 μM, 10 μM, and 20 μM to determine the effectiveness of each compound in inhibiting the proliferation of cancer cells. At the same time, a control group consisting of a normal fibroblast cell line (3T3-L1) was included in the study. The assay known as MTT [3-(4,5-dimethylthiazol-2-yl)-2,5-diphenyltetrazolium bromide] has been used to determine the extent to which cytotoxic medications reduced the ability to survive of cancer cells. Table 3 provides a summary of the estimated IC_50_ values, percent (%) inhibition, and viability of drugs tested in MDA-MB-231 cells. The dosage response was analyzed and IC_50_ values (computed by IBM SPSS Statistics 26 software). The results obtained from the MTT experiment indicate that both **MI-44** and **MI-217** exhibit significant efficacy in inhibiting the growth of MDA-MB-231 cells, as evidenced by their respective IC_50_ values of 7.4 ± 0.6 μM and 6.2 ± 0.4 μM. To ascertain the selectivity of lethal effects exhibited by compounds towards malignant cells, the non-tumorigenic 3T3-L1 cells were subjected to varying doses of both **MI-44** and **MI-217** substances i.e., 2.5 μM, 5 μM, 10 μM, and 20 μM in a similar manner like cancer cells. Table 4 presents the findings of the effects of **MI-44** and **MI-217** on 3T3-L1 cell lines. The results suggest that these cells exhibit a reduced vulnerability to the influence of both **MI-44** and **MI-217**. Notably, **MI-217** demonstrated a higher level of cell death in breast cancer cells. Based on the results of this investigation, the cytotoxicity of **MI-44** and **MI-217** was significantly higher when applied to the aggressive triple-negative MDA-MB-231 cells. These new compounds, **MI-44** and **MI-217**, may offer promising therapeutic treatment for patients with breast cancer, as exposure to them reduced cytotoxicity in non-tumorigenic 3T3-L1 cells. The inhibition of selected inhibitors is given in Tables 4.

**Table 3:** % viability and inhibition of **MI-44** and **MI-217** on Breast cancer cell line (MDA-MB-231).

Compound	Conc (μM)	%Viability	%Inhibition	IC_50_ (μM)
**MI-44**	2.5	90.57	9.43	7.4 ± 0.6
	5	55.19	44.81	
	10	40.52	59.48	
	20	15.59	84.41	
**MI-217**	2.5	87.67	12.33	6.2 ± 0.4
	5	63.22	36.78	
	10	31.31	68.69	
	20	12.43	87.57	
**Cisplatin**	2.5	92.11	7.89	18.4 ± 0.2
	5	79.43	20.57	
	10	67.51	32.49	
	20	38.17	61.83

**Table 4:** % viability and inhibition of MI-44 and MI-217 on Normal 3T3-L1 fibroblast cell line.

Compounds	Conc (μM)	%Viability	%Inhibition	IC_50_ (μM)
**MI-44**	2.5	96.5	3.5	>20
	5	93.60	6.4	
	10	83.24	16.76	
	20	78.70	21.3	
**MI-217**	2.5	92.53	7.47	>20
	5	87.36	12.64	
	10	80.82	19.18	
	20	76.33	23.67	
**Cisplatin**	2.5	94.78	5.22	>20
	5	89.58	10.42	
	10	83.01	16.99	
	20	79.11	20.89	

#### Flow cytometry analysis

**Figure 11:**
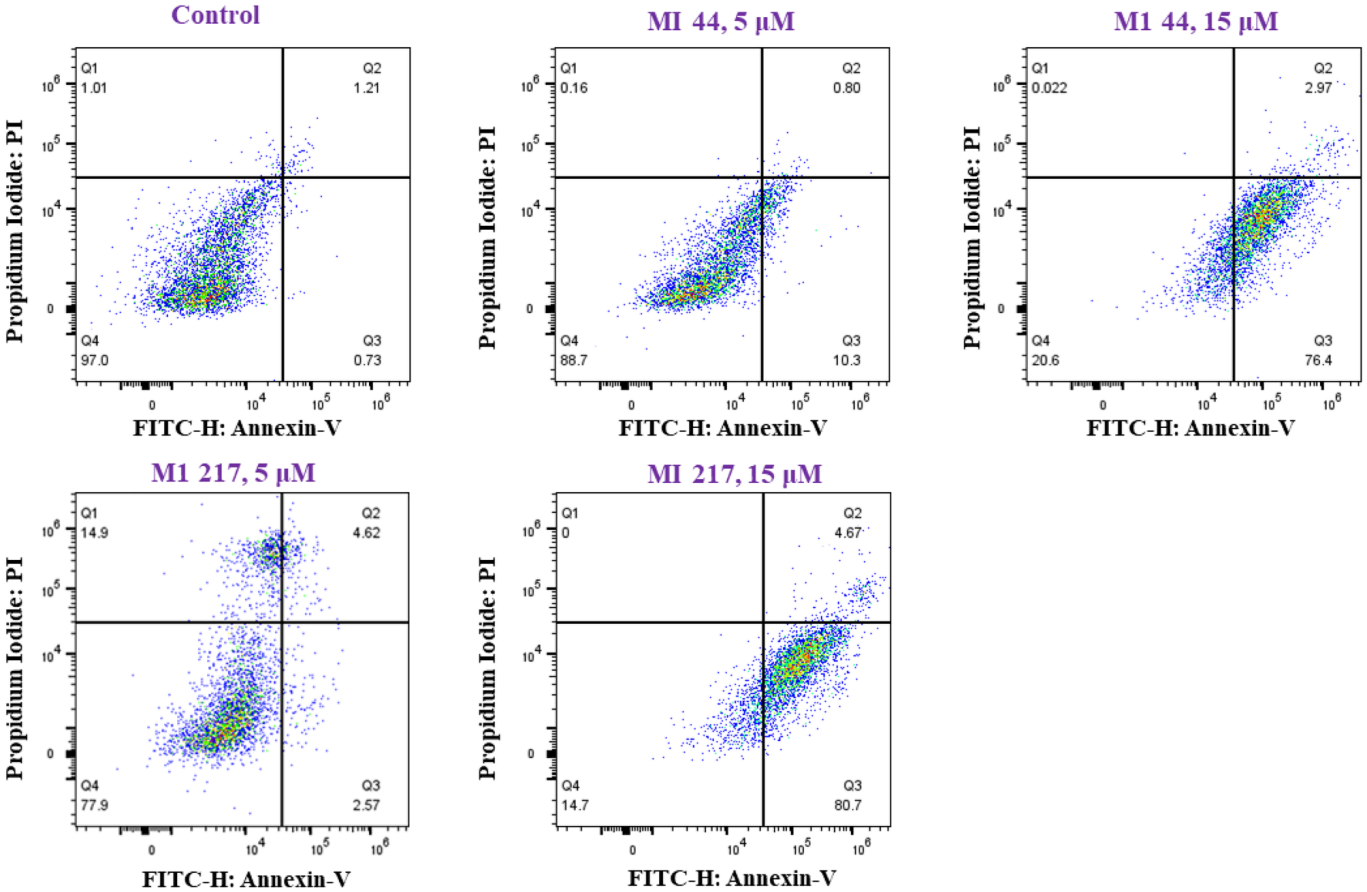
Annexin V-FITC/PI double stained cells were treated with compounds, **MI-44** and **MI-217** at 5–15 μM for 48 h. The percentage of apoptotic/necrotic cells was calculated by FlowJo_v10.9.0 software (mean ± SD, n = 3); /p* < 0.05 compared with the control.

To assess the specific types of cell death, namely apoptosis or necrosis, triggered by **M1-44** and **MI-217**, the MDA-MB-231 breast cancer cell line was subjected to a 48-h treatment with **M1-44** and **MI-217**. Subsequently, the cells were stained with Annexin V-FITC and PI, and subsequently examined using flow cytometry. As depicted in Fig. 8, the utilization of flow cytometric analysis revealed a dose-dependent reduction in the population of normal cells (Annexin V-FITC-/PI-) upon treatment with **M1-44** and **MI-217.** The population of apoptotic cells, comprising both early-stage apoptotic cells (Annexin V-FITC+/PI−) and late apoptotic cells (Annexin V-FITC+/PI+), exhibited a proportional increase in response to varying doses. Upon increasing the treatment concentrations, the proportion of normal cells exhibited a decline, dropping from 97.00% in the control group to 20.6% at a concentration of 15 μM for **MI-44**, and to 14.7% for **MI-217**. The percentage of apoptotic cells (including early apoptotic and late apoptotic) increased from 1.94% (control) to 79.37% (15 μM) for **MI-44** and to 85.37% for **MI-217 **(Fig. 11 and Table S6).

## Conclusion

The purpose of this work was to find possible inhibitors of SIRT3, which would stop deacetylation and hinder the proliferation of breast cancer cells. We have identified eight potential drug-like candidates for SIRT3 through extensive computational drilling, which exhibited excellent docking scores, binding affinities, and strong interactions with SIRT3, however, 2/8 molecules (**MI-44** and **MI-217**) possess favorable pharmacokinetics and drug-likeness in the in-silico analysis, and outstanding pharmacokinetics/dynamics profile. MD simulations were used to further optimize and enhance the docking contact of these two compounds. Compounds MI-44 and MI-217 showed the highest energy values, with values of − 45.61 ± 0.064 kcal/mol and − 41.65 ± 0.089 kcal/mol, as well, according to the results of binding free energy simulations. These results imply that these chemicals and the SIRT3 catalytic domain have a strong binding compatibility. Therefore, their inhibitory potential was tested in breast cancer cell line (MDA-MB-231) through MTT assay, and their ability to cause apoptosis of cancer cells was determined by flow cytometry. The percentage of apoptotic cells (including early and late apoptotic cells) increased from 1.94% in control to 79.37% for **MI-44** and 85.37% for **MI-217** at 15 μM. Apoptotic cell death was effectively induced by these two compounds in a flow cytometry assay indicating them as a good inhibitor of human SIRT3. All things considered, this work offers insightful information on the identification and improvement of substances that could function as potent therapeutic agents for SIRT3, presenting encouraging possibilities for the therapy of breast cancer.

### Supplementary Information

Supplementary Information.

## Data Availability

All data generated or analysed during this study are included in this published article [and its supplementary information files].
