# Supplementary material for: Identification of small molecular inhibitors of SIRT3 by computational and biochemical approaches a potential target of breast cancer
Source: Sci Rep. 2024 May 30;14:12475. doi: 10.1038/s41598-024-63177-7 (PMC11139978; doi:10.1038/s41598-024-63177-7)
Supplement: Supplementary file 1 — Supplementary Information. [file 41598_2024_63177_MOESM1_ESM.zip › Supplemantary material file 1.docx]

**Identification of small molecular inhibitors of SIRT3 by computational and biochemical approaches a potential target of breast cancer**

Atta Ullah^1^†, Najeeb Ur Rehman^1^†, Waseem Ul Islam^2^, Faizullah Khan^1^, Muhammad Waqas^1^, Sobia Ahsan Halim^1^*, Afnan Jan^3^, Abdullatif Bin Muhsinah^4^, Ajmal Khan^1^*, Ahmed Al-Harrasi^1^*

^1^Natural and Medical Sciences Research Center, University of Nizwa, PO Box 33, 616 Birkat Al Mauz, Nizwa, Oman; [aharrasi@unizwa.edu.om](mailto:aharrasi@unizwa.edu.om) (A.A-H)

^2^Department of Pharmacy, University of Swabi, Khyber Pakhtunkhwa, Pakistan [waseemkkhan1212@gmail.com](mailto:waseemkkhan1212@gmail.comharrasi@unizwa.edu.om) (W.I)

^3^Umm Al-Qura University, Faculty of Medicine, Department of Biochemistry, Makkah, Kingdom of Saudi Arabia. [Amjan@uqu.edu.sa](mailto:Amjan@uqu.edu.sa) (A.J.)

^4^Department of Pharmacognosy, College of Pharmacy, King Khalid University, Abha 61441, Saudi Arabia; [Ajmohsnah@kk.edu.sa](mailto:Ajmohsnah@kk.edu.sa) (A.B. M)

† equal contribution

*Correspondence; [sobia_halim@unizwa.edu.om](mailto:sobia_halim@unizwa.edu.om) (S.A.H); [ajmalkhan@unizwa.edu.om](mailto:ajmalkhan@unizwa.edu.om) (A.K); [aharrasi@unizwa.edu.om](mailto:aharrasi@unizwa.edu.om) (A.A-H)

**Table of content**

| **Table S1** | Molecular interactions between EX527 at SIRT3 active site in PDB ID: 4BVH |
| --- | --- |
| **Table S2** | PPI network of SIRT3 showing the K-mean clustering method, node identifier, node degree and position obtained through server STRING. |
| **Table S3** | The docking interaction of selected compounds within SIRT3 is tabulated. |
| **Table S4** | Pharmacokinetics properties of selected compounds **MI-212-M2-cdx**. |
| **Table S5** | Hydrogen bond analysis of selected compounds in a 300 ns simulation trajectory. |
| **Table S6** | The percentage of live, early, and late appoptosis for **MI-44** and **MI-217** |
| **Figure S1** | Redocking of co-crystalized ligand (Ex-527) of human SIRT3 PDB ID: 4BVH. The Red color show the ligand and green color show the redock position with an RMSD value of 0.9272 Å*.* |
| **Figure S2** | Hydrogen bond analysis of selected inhibitors compounds (MI-44 and MI-217) against human SIRT 3 PDB ID: 4BVH. |

**Table S1.** Molecular interactions between EX527 at SIRT3 active site in PDB ID: 4BVH

| **Name** | **Ligand** | **Receptor** | **Interaction** | **Distance (Å)** | **E (kcal/mol)** |
| --- | --- | --- | --- | --- | --- |
| **Ex-527** | N | ASP231 | HBD | 2.89 | -5.0 |
|  | O | ILE230 | HBA | 2.78 | -3.1 |
|  | O | ASP231 | HBA | 3.17 | -0.6 |
|  | 5-ring | ILE230 | π-H | 4.14 | -0.8 |
|  | 5-ring | ILE230 | π-H | 3.69 | -0.6 |
| ***HBA****= Hydrogen bond acceptor,* ***HBD=*** *Hydrogen bond doner,* ***π*-H**= Pi-Hydrogen | | | | | |

**Table S2.** PPI network of SIRT3 showing the K-mean clustering method, node identifier, node degree and position obtained through server STRING.

| **clustering method** | **cluster number** | **cluster color** | **gene count** | **protein name** |
| --- | --- | --- | --- | --- |
| k-means | 1 | Red | 5 | MDM2 |
| k-means | 1 | Red | 5 | MDM4 |
| k-means | 1 | Red | 5 | PTGS2 |
| k-means | 1 | Red | 5 | SIRT1 |
| k-means | 1 | Red | 5 | TP53 |
| k-means | 2 | Green | 4 | IDH2 |
| k-means | 2 | Green | 4 | SIRT3 |
| k-means | 2 | Green | 4 | SOD2 |
| k-means | 2 | Green | 4 | SOD2-2 |
| k-means | 3 | Blue | 1 | GPX4 |
|  | | | | |
| **node** | **identifier** | **X-position** | **Y-position** | **color** |
| GPX4 | 9606.ENSP00000346103 | 0.65 | 0.21 | rgb(255,175,101) |
| IDH2 | 9606.ENSP00000331897 | 0.46 | 0.78 | rgb(150,101,255) |
| MDM2 | 9606.ENSP00000258149 | 0.35 | 0.56 | rgb(0,28,178) |
| MDM4 | 9606.ENSP00000356150 | 0.36 | 0.8 | rgb(178,171,0) |
| PTGS2 | 9606.ENSP00000356438 | 0.52 | 0.16 | rgb(187,255,101) |
| SIRT1 | 9606.ENSP00000212015 | 0.45 | 0.36 | rgb(101,199,255) |
|  | | | | |
| **node** | **identifier** | **node degree** |  | |
| GPX4 | 9606.ENSP00000346103 | 5 |  |  |
| IDH2 | 9606.ENSP00000331897 | 7 |  |  |
| MDM2 | 9606.ENSP00000258149 | 6 |  |  |
| MDM4 | 9606.ENSP00000356150 | 3 |  |  |
| PTGS2 | 9606.ENSP00000356438 | 6 |  |  |
| SIRT1 | 9606.ENSP00000212015 | 8 |  |  |
| SIRT3 | 9606.ENSP00000372191 | 6 |  |  |
| SOD2 | 9606.ENSP00000446252 | 6 |  |  |
| SOD2-2 | 9606.ENSP00000442920 | 6 |  |  |
| TP53 | 9606.ENSP00000269305 | 9 |  |  |

**Table S3.** The docking interaction of selected compounds within SIRT3 is tabulated.

| **Compounds codes** | **Docking**  **score (kcal/mol)** | **Ligand**  **Atoms** | **Receptor**  **Atoms** | **Bond type** | **Bond**  **Distance (Å)** | **Energy (kcal/mol)** |
| --- | --- | --- | --- | --- | --- | --- |
| **MI-212** | -8.37 | N4 | ASN229 | HBD | 3.01 | -1.1 |
|  |  | S6 | ALA146 | HBD | 3.41 | -1.5 |
|  |  | S6 | ASP231 | HBD | 3.30 | -1.2 |
|  |  | S6 | SER149 | HBA | 3.56 | -0.5 |
| **MI-217** | -8.15 | N4 | ASN229 | HBD | 2.91 | -4.3 |
|  |  | C5 | ASP231 | HBD | 3.30 | -0.6 |
|  |  | S6 | ALA146 | HBD | 3.31 | -1.7 |
|  |  | S6 | PRO155 | HBD | 3.52 | -1.2 |
|  |  | S6 | ASN229 | HBD | 3.32 | -11.3 |
|  |  | N7 | PRO155 | HBD | 2.74 | -0.7 |
| **MI-210** | -8.06 | N4 | ASN229 | HBD | 2.93 | -3.3 |
|  |  | 6-ring | PHE157 | H-pi | 3.84 | -0.6 |
| **MI-257** | 7.95 | N11 | ASN229 | HBD | 2.95 | -2.4 |
|  |  | N3 | ILE230 | HBA | 3.36 | -1.9 |
|  |  | S13 | ALA146 | HBA | 3.93 | -0.6 |
|  |  | S13 | SER149 | HBA | 4.18 | -0.5 |
| **MI-202** | -7.93 | N4 | ASN229 | HBD | 2.97 | -2.1 |
|  |  | N7 | PRO155 | HBA | 3.03 | -3.0 |
|  |  | S6 | SER149 | HBA | 3.99 | -0.6 |
| **MI-44** | -7.48 | N10 | ASP231 | HBD | 2.75 | -4.3 |
|  |  | N12 | ALA146 | HBD | 2.62 | -9.9 |
|  |  | S13 | ALA146 | HBD | 3.29 | -1.1 |
|  |  | N3 | PRO155 | HBD | 3.36 | -1.0 |
| **MI-84** | -7.44 | N6 | PRO155 | HBD | 2.84 | -2.0 |
|  |  | S5 | ILE230 | HBA | 3.64 | -1.6 |
|  |  | N7 | GLN228 | HBD | 2.99 | -0.8 |
| **M2-cdx** | -7.31 | N13 | ASP231 | HBD | 2.55 | -7.3 |
|  |  | N14 | ILE230 | HBA | 3.03 | -3.7 |
|  |  | 5-ring | HIS248 | π-H | 4.64 | -0.5 |
| ***HBA****= Hydrogen bond acceptor,* ***HBD=*** *Hydrogen bond doner,* ***π-H*** *= Pi-hydrogen bond* | | | | | | |

**Table S4**. Pharmacokinetics properties of selected compounds **MI-212-M2-cdx**.

| **Compounds** | **GIA** | **BBBP** | **P-gp substrate** | **CYP1A2 inhibitor** | **CYP2C19 inhibitor** | **CYP2C9 inhibitor** | **CYP2D6 inhibitor** | **Lipinski** | **Ghose** | **Muegge** |
| --- | --- | --- | --- | --- | --- | --- | --- | --- | --- | --- |
| **MI-212** | High | No | No | Yes | Yes | Yes | No | Yes | Yes | Yes |
| **MI-217** | High | No | No | Yes | No | No | No | Yes | Yes | Yes |
| **MI-210** | High | No | No | Yes | Yes | No | No | Yes | Yes | Yes |
| **MI-257** | High | No | No | Yes | Yes | Yes | No | Yes | Yes | Yes |
| **MI-202** | High | No | No | Yes | Yes | Yes | No | Yes | Yes | Yes |
| **MI-44** | High | No | No | Yes | No | No | No | Yes | Yes | Yes |
| **MI1-84** | High | No | No | Yes | No | No | No | Yes | Yes | Yes |
| **M2-cdx** | High | No | No | Yes | No | No | No | Yes | Yes | Yes |
| ***GIA****= Gastrointestinal absorption,* ***BBBP****= Blood brain barrier penetration,* ***P-gp*** *= P-glycoprotein* | | | | | | | | | | |

**Table S5.** Hydrogen bond analysis of selected compounds in a 300 ns simulation trajectory.

| **Compounds** | **Acceptor Atoms** | **Donor Atoms** | **Bond Life** | **Distance (Å)** | **Angle (Å)** |
| --- | --- | --- | --- | --- | --- |
| **4BVH** | OCZ_279-OAP | ILE_116-N | 91.24% | 2.83 | 163.32 |
|  | COZ_1-O1A | SER_207-OG | 75.38% | 2.64 | 164.67 |
|  | COZ_1-O2B | THR_206-OG1 | 63.27% | 2.61 | 163.23 |
|  | ASP_117-OD2 | OCZ_279-NAQ | 63.24% | 2.86 | 165.26 |
|  | COZ_1-O2B | SER_207-N | 46.07% | 2.81 | 152.29 |
|  | COZ_1-N1 | VAL_252-N | 33.74% | 2.91 | 154.90 |
|  | COZ_1-O2A | ARG_44-NE | 25.23% | 2.85 | 156.62 |
|  | COZ_1-O2A | ARG_44-NH2 | 23.90% | 2.81 | 156.72 |
|  | COZ_1-O2B | LEU_208-N | 20.78% | 2.88 | 156.45 |
|  | GLN_114-O | OCZ_279-NAG | 16.43% | 2.88 | 150.00 |
|  | COZ_1-O3' | ASN_230-ND2 | 13.04% | 2.91 | 161.08 |
|  | COZ_1-O1B | THR_206-OG1 | 12.61% | 2.63 | 165.85 |
|  | COZ_1-N7 | THR_36-OG1 | 10.39% | 2.85 | 158.45 |
|  | THR_36-OG1 | COZ_1-N6 | 9.99% | 2.90 | 156.94 |
|  | ASP_232-OD1 | COZ_1-O2' | 9.99% | 2.73 | 157.07 |
|  | COZ_1-O2B | ALA_32-N | 9.77% | 2.86 | 160.48 |
|  | THR_36-OG1 | COZ_1-N6 | 8.82% | 2.90 | 156.92 |
|  | COZ_1-O5D | GLN_114-NE2 | 8.79% | 2.86 | 151.41 |
|  | ASP_232-OD2 | COZ_1-O2' | 8.74% | 2.72 | 158.08 |
|  | COZ_1-O1B | SER_207-N | 8.35% | 2.86 | 154.86 |
|  | COZ_1-O1A | SER_207-N | 6.79% | 2.89 | 146.28 |
|  | COZ_1-O1A | ARG_44-NH1 | 6.10% | 2.75 | 157.17 |
|  | COZ_1-O4D | ARG_44-NH1 | 5.69% | 2.83 | 157.18 |
|  | GLN_114-OE1 | COZ_1-O3D | 5.37% | 2.72 | 164.40 |
|  | ASN_230-OD1 | COZ_1-O3' | 5.16% | 2.75 | 159.55 |
|  | ASN_230-OD1 | COZ_1-O2' | 5.07% | 2.76 | 155.86 |
|  | OCZ_279-OAP | ASP_117-N | 5.04% | 2.93 | 148.80 |
|  | COZ_1-N3 | ARG_231-N | 4.86% | 2.94 | 151.52 |
|  | COZ_1-O3D | ARG_44-NH2 | 4.73% | 2.88 | 160.96 |
|  | COZ_1-O2A | ARG_44-NH2 | 4.64% | 2.81 | 156.06 |
|  | COZ_1-O4D | ARG_44-NH2 | 4.55% | 2.86 | 152.73 |
|  | ASP_232-OD2 | COZ_1-O3' | 3.65% | 2.68 | 162.95 |
|  | COZ_1-O2' | ASN_230-ND2 | 3.59% | 2.89 | 154.73 |
|  | COZ_1-O1B | ARG_44-NH1 | 3.58% | 2.80 | 155.67 |
|  | COZ_1-O1A | ARG_44-NH2 | 3.58% | 2.86 | 152.03 |
|  | COZ_1-O1D | TYR_51-OH | 3.58% | 2.81 | 158.46 |
|  | COZ_1-O2A | ARG_44N | 3.47% | 2.88 | 165.63 |
|  | COZ_1-O2A | ARG_44-NH1 | 3.30% | 2.79 | 155.72 |
|  | COZ_1-O1B | ARG_44-NH2 | 3.30% | 2.80 | 153.94 |
|  | COZ_1-O1B | ARG_44-NH2 | 3.05% | 2.82 | 158.95 |
|  | ASP_232-OD1 | COZ_1-O3' | 2.96% | 2.67 | 164.13 |
|  | COZ_1-O2B | ARG_44-NH1 | 2.80% | 2.81 | 158.81 |
|  | COZ_1-N6 | ARG_231-NH2 | 2.66% | 2.91 | 156.54 |
|  | ASP_251-OD2 | COZ_1-N6 | 2.65% | 2.87 | 158.05 |
|  | ASP_251-OD1 | COZ_1-N6 | 2.50% | 2.87 | 158.46 |
|  | COZ_1-O6D | HID_134-ND1 | 2.17% | 2.83 | 145.46 |
|  | COZ_1-O2B | ARG_44-NH2 | 1.93% | 2.81 | 158.77 |
|  | COZ_1-N6 | THR_36-OG1 | 1.50% | 2.85 | 161.51 |
|  | ASP_251-OD2 | COZ_1-N6 | 1.25% | 2.86 | 160.29 |
|  | COZ_1-O1D | ARG_44-NH2 | 1.15% | 2.90 | 153.80 |
|  | COZ_1-O1D | HID_134-ND1 | 1.06% | 2.91 | 143.84 |
| **MI-44** | MI44_1-O1A | ARG_44-NH1 | 97.01% | 2.77 | 159.24 |
|  | MI44_1-O1B | SER_207-N | 74.80% | 2.86 | 158.74 |
|  | MI44_1-O2B | ALA_32-N | 63.44% | 2.86 | 159.94 |
|  | MI44_1-O1B | SER_207-OG | 50.52% | 2.65 | 164.52 |
|  | MI44_1-O2A | PHE_43-N | 48.15% | 2.86 | 146.96 |
|  | MI44_1-O1A | SER_207-OG | 47.78% | 2.66 | 163.86 |
|  | MI44_1-O1B | THR_206-OG1 | 46.51% | 2.71 | 161.61 |
|  | MI44_1-O4D | ARG_44-NH1 | 43.39% | 2.87 | 148.57 |
|  | ASN_230-OD1 | MI44_1-O3' | 43.25% | 2.76 | 160.62 |
|  | MI44_1-O6D | TYR_51-OH | 42.70% | 2.73 | 158.74 |
|  | MI44_1-O2B | THR_206-OG1 | 42.18% | 2.75 | 166.85 |
|  | MI44_1-N1 | VAL_252-N | 38.66% | 2.91 | 153.86 |
|  | MI44_1-O2A | ARG_44-N | 38.38% | 2.89 | 163.65 |
|  | MI44_1-O4D | ARG_44-NH2 | 29.41% | 2.89 | 149.34 |
|  | MI44_1-O3' | ASN_230-ND2 | 21.96% | 2.89 | 155.62 |
|  | ASN_115-OD1 | MI44_279-N4 | 18.62% | 2.90 | 149.75 |
|  | MI44_1-O2B | GLN_114-NE2 | 15.88% | 2.86 | 146.81 |
|  | MI44_1-O5D | ARG_44-NH1 | 15.79% | 2.86 | 142.34 |
|  | MI44_1-O1D | TYR_51-OH | 15.65% | 2.82 | 152.61 |
|  | ASN_230-OD1 | MI44_1-O2' | 14.86% | 2.77 | 157.69 |
|  | MI44_1-O1D | ARG_44-NH2 | 12.46% | 2.88 | 145.71 |
|  | THR_36-OG1 | MI44_1-N6 | 10.25% | 2.90 | 159.34 |
|  | ASP_232-OD2 | MI44_1-O2' | 8.53% | 2.71 | 162.51 |
|  | THR_36-OG1 | MI44_1-N6 | 8.27% | 2.90 | 159.67 |
|  | ASP_232-OD1 | MI44_1-O2' | 7.41% | 2.71 | 161.79 |
|  | MI44_1-N3 | ARG_231-N | 5.23% | 2.94 | 147.90 |
|  | MI44_1-N7 | THR_36-OG1 | 4.35% | 2.86 | 157.93 |
|  | ASP_251-OD2 | MI44_1-N6 | 3.79% | 2.86 | 161.07 |
|  | MI44_1-N6 | ARG_231-NH2 | 3.10% | 2.91 | 152.92 |
|  | ASP_251-OD2 | MI44_1-N6 | 3.06% | 2.86 | 160.69 |
|  | ASP_251-OD1 | MI44_1-N6 | 3.04% | 2.86 | 160.97 |
|  | MI44_1-O2' | ARG_231-N | 2.74% | 2.92 | 151.41 |
|  | ASP_251-OD1 | MI44_1-N6 | 2.53% | 2.86 | 161.99 |
|  | PRO_41-O | MI44_279-N7 | 2.37% | 2.92 | 156.28 |
|  | MI44_1-O3D | HID_134-ND1 | 1.85% | 2.87 | 142.23 |
|  | GLN_114-OE1 | MI44_1-O3D | 1.41% | 2.74 | 164.80 |
|  | ASP_232-OD1 | MI44_1-O3' | 1.37% | 2.69 | 163.25 |
|  | ASP_232-OD2 | MI44_1-O3' | 1.25% | 2.69 | 163.71 |
|  | MI44_1-N1 | ARG_231-NH2 | 1.16% | 2.90 | 149.96 |
| **MI-217** | MI127_1O1A | ARG_44NH1 | 97.51% | 2.76 | 159.36 |
|  | MI127_1O1B | THR_206OG1 | 92.46% | 2.69 | 163.96 |
|  | MI127_1O1A | SER_207OG | 89.54% | 2.67 | 162.91 |
|  | MI127_1O2B | ALA_32N | 81.45% | 2.85 | 161.03 |
|  | MI127_1O1B | SER_20N | 66.09% | 2.87 | 156.88 |
|  | MI127_1O1D | TYR_51OH | 61.19% | 2.80 | 158.74 |
|  | ASN_230OD1 | MI127_1O3' | 50.86% | 2.76 | 160.82 |
|  | MI127_1O2A | PHE_43N | 49.44% | 2.88 | 148.35 |
|  | MI127_1O4D | ARG_44NH1 | 48.94% | 2.87 | 150.17 |
|  | ASN_115OD1 | MI127_279N4 | 47.67% | 2.86 | 152.08 |
|  | MI127_1O2A | ARG_44N | 45.70% | 2.89 | 164.77 |
|  | MI127_1O2B | GLN_114NE2 | 41.57% | 2.83 | 147.72 |
|  | MI127_1N1 | VAL_252N | 38.60% | 2.91 | 153.12 |
|  | MI127_1O4D | ARG_44NH2 | 16.99% | 2.90 | 148.24 |
|  | MI127_1O1D | ARG_44NH2 | 13.01% | 2.89 | 149.53 |
|  | MI127_1O3' | ASN_230ND2 | 11.50% | 2.89 | 156.46 |
|  | THR_36OG1 | MI127_1N6 | 11.04% | 2.90 | 159.15 |
|  | ASN_230OD1 | MI127_1O2' | 8.86% | 2.78 | 157.82 |
|  | PRO_41O | MI127_279N7 | 7.71% | 2.82 | 153.40 |
|  | ASP_117OD2 | MI127_279N4 | 7.16% | 2.72 | 155.33 |
|  | MI127_1O1B | SER_207OG | 7.08% | 2.72 | 159.75 |
|  | ASP_117OD1 | MI127_279N7 | 6.81% | 2.81 | 148.13 |
|  | THR_36OG1 | MI127_1N6 | 6.47% | 2.90 | 159.47 |
|  | MI127_1N7 | THR_36OG1 | 5.94% | 2.84 | 159.26 |
|  | ASP_251OD2 | MI127_1N6 | 5.89% | 2.87 | 161.20 |
|  | ASP_251OD1 | MI127_1N6 | 5.65% | 2.87 | 161.46 |
|  | MI127_1O5D | ARG_44NH1 | 5.64% | 2.85 | 140.85 |
|  | ASP_117OD2 | MI127_279N7 | 5.06% | 2.82 | 147.22 |
|  | MI127_1N6 | ARG_231NH2 | 4.29% | 2.91 | 154.16 |
|  | GLN_114OE1 | MI127_1O3D | 4.12% | 2.73 | 162.53 |
|  | MI127_1O2' | ARG_231N | 3.18% | 2.91 | 154.89 |
|  | MI127_N3 | ARG_231N | 2.89% | 2.94 | 147.02 |
|  | MI127_1O2B | THR_206OG1 | 2.77% | 2.77 | 166.34 |
|  | ASP_251OD2 | MI127_1N6 | 2.44% | 2.86 | 160.53 |
|  | ASP_251OD1 | MI127_1N6 | 1.99% | 2.87 | 161.44 |
|  | ASP_232OD2 | MI127_1O2' | 1.66% | 2.71 | 160.86 |
|  | ASP_232OD1 | MI127_1O2' | 1.57% | 2.70 | 161.86 |
|  | MI127_1O5' | SER_207N | 1.37% | 2.92 | 141.50 |
|  | MI127_1O2' | ARG_231NH1 | 1.30% | 2.89 | 149.07 |

**Table S6**. The percentage of live, early, and late appoptosis for **MI-44** and **MI-217**

| **Percentage of cells** | **Control** | **MI-44**  **(5µM)** | **MI-44**  **(15µM)** | **MI-217**  **(5µM)** | **MI-217**  **(15µM)** |
| --- | --- | --- | --- | --- | --- |
| Live cell | 97 | 88.7 | 20.6 | 77.9 | 14.7 |
| Early apoptosis | 0.73 | 10.3 | 76.4 | 2.57 | 80.7 |
| Late apoptosis | 1.21 | 0.80 | 2.97 | 4.62 | 4.57 |

**Figure S1.** Redocking of co-crystalized ligand (Ex-527) of human SIRT3 PDB ID: 4BVH. The Red color show the ligand and green color show the redock position with an RMSD value of 0.9272 Å.

**Figure S2.** Hydrogen bond analysis of selected inhibitors compounds (MI-44 and MI-217) against human SIRT 3 PDB ID: 4BVH.
